# Inhibition of BRD4 attenuates tumor cell self-renewal and suppresses stem cell signaling in MYC driven medulloblastoma

**DOI:** 10.18632/oncotarget.1659

**Published:** 2014-03-31

**Authors:** Sujatha Venkataraman, Irina Alimova, Ilango Balakrishnan, Peter Harris, Diane K Birks, Andrea Griesinger, Vladimir Amani, Brian Cristiano, Marc Remke, Michael D Taylor, Michael Handler, Nicholas K Foreman, Rajeev Vibhakar

**Affiliations:** ^1^ Department of Pediatrics, Children's Hospital Colorado and University of Colorado, Denver, CO, USA; ^2^ Department of Neurosurgery, Children's Hospital Colorado and University of Colorado, Denver, CO, USA; ^3^ Division of Neurosurgery, Program in Developmental and Stem Cell Biology, Hospital for Sick Children, Toronto, ON, Canada

**Keywords:** Medulloblastoma, MYC, BRD4, JQ1, Senescence

## Abstract

Medulloblastoma is a pediatric brain tumor with a variable prognosis due to clinical and genomic heterogeneity. Among the 4 major genomic sub-groups, patients with MYC amplified tumors have a particularly poor prognosis despite therapy with surgery, radiation and chemotherapy. Targeting the MYC oncogene has traditionally been problematic. Here we report that MYC driven medulloblastoma can be targeted by inhibition of the bromodomain protein BRD4. We show that bromodomain inhibition with JQ1 restricts c-MYC driven transcriptional programs in medulloblastoma, suppresses medulloblastoma cell growth and induces a cell cycle arrest. Importantly JQ1 suppresses stem cell associated signaling in medulloblastoma cells and inhibits medulloblastoma tumor cell self-renewal. Additionally JQ1 also promotes senescence in medulloblastoma cells by activating cell cycle kinase inhibitors and inhibiting activity of E2F1. Furthermore BRD4 inhibition displayed an anti-proliferative, pro-senescence effect in a medulloblastoma model *in vivo.* In clinical samples we found that transcriptional programs suppressed by JQ1 are associated with adverse risk in medulloblastoma patients. Our work indicates that BRD4 inhibition attenuates stem cell signaling in MYC driven medulloblastoma and demonstrates the feasibility BET domain inhibition as a therapeutic approach *in vivo*.

## INTRODUCTION

Medulloblastoma is the most common malignant brain tumor of children. Surgery, radiation and chemotherapy are the cornerstones of medulloblastoma therapy, with outcomes far from optimal[1]. Further, there is increasing evidence of long-term morbidity such as neurocognitive deficits and secondary tumors associated with current treatments[2]. Recent transcriptional profiling studies demonstrate that medulloblastoma is a heterogeneous disease with 4 distinct molecular subgroups characterized by unique genomic and clinical features[3–7]. Two subgroups are associated with specific abnormalities in developmental pathways; Wingless (WNT) and Sonic Hedgehog (SHH) respectively. The other two groups are less well characterized (Group 3 and 4)[8]. Among these, Group 3 patients with high c-MYC do very poorly with 5-year survival less than 30%[9, 10]. Thus, there is a critical need for more effective therapies to combat this disease, particularly in the high c-MYC expressing tumors.

MYC is a major regulatory factor of stem cell maintenance and tumor cell proliferation[11]. Importantly the c-MYC oncogene family is the most commonly amplified gene set in medulloblastoma further emphasizing the need to target these tumors[12]. While the concept of c-MYC as a therapeutic target is well established, direct targeting of c-MYC has remained problematic. For example early studies using an inducible c-MYC model showed that activation of c-MYC promoted lymphoma formation that regressed upon removal of c-MYC expression[13]. More recently, expressing a dominant-negative c-MYC allele in a KRAS- dependent murine model of lung adenocarcinoma further demonstrated the therapeutic benefit of c-MYC inhibition[14]. Similarly using G quadraplex molecules Shalaby *et al* demonstrated that inhibition of c-MYC was a potent strategy for suppressing medulloblastoma[15].

Nevertheless, a therapeutic approach to target c-MYC has remained elusive. The absence of a clear ligand-binding domain has presented a daunting obstacle toward direct inhibition of MYC. However because c-MYC is a DNA binding transcriptional activator, targeting c-MYC driven transcription provides an opportunity to suppress c-MYC driven oncogenesis. Recently inhibition of the bromodomain and extraterminal domain (BET) protein BRD4 was shown to be a key mediator of MYC driven transcriptional programs providing a therapeutic target in c-MYC driven tumors[16, 17].

The bromodomain and extraterminal domain (BET) family is composed of four members; BRD2, BRD3, BRD4, and BRDT. BET family proteins bind to acetylated histones to influence transcription[18]. BET proteins are attractive therapeutic targets given the recent description of several small molecule inhibitors including JQ1 and iBET [19–21]. Several hematologic malignancies, the highly malignant NUT midline carcinoma and the pediatric adrenal gland tumor neuroblastoma are responsive to BRD4 inhibition *in vitro* and in mouse models [16, 17, 22–24]. Furthermore two recent reports also show the utility of BRD4 inhibition in medulloblastoma[25, 26].

Here we show that BRD4 inhibition is a highly effective strategy to inhibit MYC driven medulloblastoma. We demonstrate that inhibition of BRD4 results in suppression of tumor cell self-renewal, stem cell signaling, and induction of senescence *in vitro* and *in vivo*.

## MATERIALS AND METHODS

### Cell lines and primary patient samples and reagents

The Daoy and D283 medulloblastoma cell lines were purchased from American Type Cell Culture (Rockville, MD). The ONS-76 medulloblastoma cell line was kindly provided by Dr. James T. Rutka (University of Toronto, Canada). D425 and D458 cell lines were kindly provided by Dr. Darell D. Bigner (Duke University Medical Center, NC). The UW228 cell line was provided by Dr. John Silber, University of Washington, Seattle, WA. Cell lines were cultured in DMEM (Gibco, Carlsbad, CA) supplemented with 10% fetal bovine serum (Atlanta Biologicals, Lawrenceville, GA).

Primary patient samples were obtained from Children's Hospital Colorado and were conducted in accordance with local and federal human research protection guidelines and Institutional Review Board (IRB) regulations. Informed consent was obtained for all specimens collected. Normal brain tissue was collected from autopsy and purchased from Ambion (Austin, TX), Stratagene (Santa Clara, CA) and Clontech Laboratories, Inc. (Mountain View, CA).

JQ1 was a kind gift from the Bradner laboratory.

### Gene expression microarray analysis

Ribonucleic acid from DMSO or JQ1 treated Daoy cells was extracted using an RNeasy kit (Qiagen, Valencia, CA) and hybridized to HG-ST2.0 Gene Chips (Affymetrix, Santa Clara, CA) according to the manufacturer's instructions and previously described by us. Data analysis was performed in R (http://www.r-project.org/), using packages publicly available through Bioconductor (http://www.bioconductor.org). Hierarchical clustering was performed using the normalized gene expression data. Heatmap visualization was performed using GENE-E (http://www.broadinstitute.org/). Functional annotation analysis of differentially expressed genes was performed with the National Institutes of Health Database for Annotation, Visualization, and Integrated Discovery (DAVID) Web tool (http://david.abcc.ncifcrf.gov/), using Biological Process Gene Ontology (GO) terms and Kyoto Encyclopedia of Genes and Genomes (KEGG) pathways. Gene set enrichment analysis (GSEA) was used to examine enrichment of genes in predefined reference sets that are based on biological knowledge using tools available from the Broad Institute (http://www.broadinstitute.org/gsea/msigdb) [27]. Unlike other approaches that examine only genes meeting a predetermined cutoff, GSEA computes an aggregate score for all genes in the reference set, based on their relative ranking in the data[27]. K-Means clustering was performed using R2 (http://hgserver1.amc.nl/cgi-bin/r2/main.cgi) in a previously published gene expression profiling cohort analyzing 199 primary medulloblastoma samples[5]. Survival differences according to clustering-derived subgroups were analyzed using Kaplan-Meier estimates and tested using Log-Rank test. P-values below 0.05 were considered to be significant.

### Cell Growth assays

Cell growth was measured using the xCELLigence system and E-Plate 96 well gold-coated plates (Roche/ACEA). This system gives the real time measurement of cell proliferation. Cells were plated on to an E-plate (1000 cells/well) allowed to grown and treated with DMSO or JQ1 24 hours later and cell growth was measured over time. Cell numbers were measured using the Viacount assay (Millipore) on a Guava Flow cytometer per the manufacturers recommendation. Briefly, cells were seeded in media and DMSO or JQ1 added 24 hours later. Cells were cultured for a further 48 hours, and collected by centrifugation. Cells were washed, Viacount reagent added and cell counts measured on the cytometer.

For the colony formation assay, cells were transfected with a shRNA for 48 hours and then plated at 500 cells per well of a 6-well plate in triplicate. After seven days of growth, the medium was aspirated, the wells were washed with PBS, and the colonies were stained with 0.5% crystal violet/25% methanol solution. The number of colonies per well was counted using a dissecting microscope with a threshold of 50 cells necessary to constitute a colony.

For methyl cellulose colony growth assays D283, D425 and D458 (500 cells/well) cells were suspended in DMEM having methyl cellulose (1.3% final) and containing either JQ1 (150 or 300nM) or DMSO control. This suspension was then plated in triplicate onto 6-well plates. The colonies were allowed to form for 8–11 days. The colonies were then stained with NBT (1mg/ml of nitro blue tetrazolium) and incubated for 24 hours at 37°C. The blue colonies with 50 or more cells in each colony were counted. Pictures were taken with GelcountTM (Oxford Optronix).

### Cell cycle assay

Flow cytometric analysis was performed to define the cell cycle distribution. Cells were seeded in 6-well plates (1×10^**5**^ cells/well) and 24 hours later were treated with JQ1. Cells were harvested 48 hours later by trypsinization and fixed with 70% ethanol overnight. Collected cells were treated with 250μl cell cycle reagent (Millipore) and evaluated per the manufacturer's recommendations.

### Real-Time Quantitative Reverse Transcriptase PCR

cDNA was generated per the manufacturer's instructions via reverse transcription using mRNA and the Mastercycler^®^ personal (Eppendorf) thermal cycler. Real-time quantitative PCR was performed with the StepOne Plus detection system (Applied Biosystems). Taqman reagents (Applied Biosystems) were used per the manufacturer's recommendations. Gene expression was determined by the ΔΔCt method. All assays were performed in triplicate.

### Western Blotting

Protein lysates were obtained from samples using RIPA buffer (Thermo Scientific, Rockford, IL) with protease inhibitors added. Western blotting was performed per standard methods. Antibodies for c-MYC and Tubulin were purchased from Cell Signaling Technology (Danvers, MA). Secondary antibodies conjugated to horseradish-peroxidase were used in conjunction with a chemiluminescent reagent to visualize protein bands.

### Luciferase reporter analysis

Luciferase assays were performed using the Cignal Pathway Reporter System (SA Biosciences) following the manufacturer's instructions. Cells were seeded into 24-well plates and transfected with luciferase reporters using Surefect transfection reagent; 8 hours later DMSO or JQ1 was added and cells incubated for an additional 24 hours. Luciferase activity was measured using the Dual Luciferase Assay system (Promega) on a Glomax multi luminometer (Promega). Firefly luciferase served as the experimental reporter and Renilla luciferase as the normalizing reporter.

### In vivo xenograft analysis

This study was conducted at Washington Biosciences Inc. at its AAALAC accredited facility. The study was conducted following industry standards including compliance to USDA and NIH animal care and use guidelines. Athymic nude mice were selected for this study because they have been used for similar tumor grafting studies and have been well-characterized. Daoy cell pellets were prepared using standard harvest procedures, resuspended in PBS and 1.0 × 10^**7**^ cells were injected into the flank of each mouse in an approximately 200 uL volume. Once tumors were palpable and measurable (mean 52 days post inoculation) treatment with JQ1 or control vehicle of DMSO was begun.

DMSO or JQ1 (50mg/kg) was dosed for 5 days a week for 4 weeks by intraperitoneal injection (n=10). Each animal was tracked individually for tumor growth by external caliper measurements of subcutaneous protruding tumor and an approximate tumor volume was calculated using the ellipsoid volume formula: π/6 × L × W × H. Animals were also weighed 3 times a week for the duration of the treatment.

### Slice culture method for BrdU and cell viability with LDH measurement

Slice cultures from primary tumor samples were grown onto Millicell Culture insert (Millipore Cat# PICM0RG50) according to manufacturer's instruction. Briefly, ~0.33cm chunks of primary tumor samples were placed onto cell culture insert and were grown in slice culture medium (Neurobasal A media containing B27, glutamax, L-glutamine, HEPES and FGF) for 5 days. On day 5, cultures were treated with, DMSO, JQ1 (300nM) or mytomycin C (50ug/mL) and fresh media with drugs was changed every day. Seven days after drug treatment, BrdU (BrdU Flow kit: BD Pharmingen Cat# 559619) was added. Two days after BrdU addition, cells and media were collected for BrdU and LDH assay. BrdU incorporation was analyzed by flow cytometry. For cytotoxicity measurement and LDH assay, Cytoscan-LDH Cytotoxicity Assay Kit- G Biosciences (Cat #786-210) was used. Briefly, media was collected from every time we changed the media on the culture and are stored at −80°C. Media was thawed and followed manufacturer's protocol to measure LDH activity that was normalized to mytomycin C treatment. Drug cytotoxicity and LDH activity was measured calorimetrically.

## RESULTS

### Bromodomain inhibition with JQ1 restricts c-MYC driven transcriptional programs in Medulloblastoma

**Figure 1:**
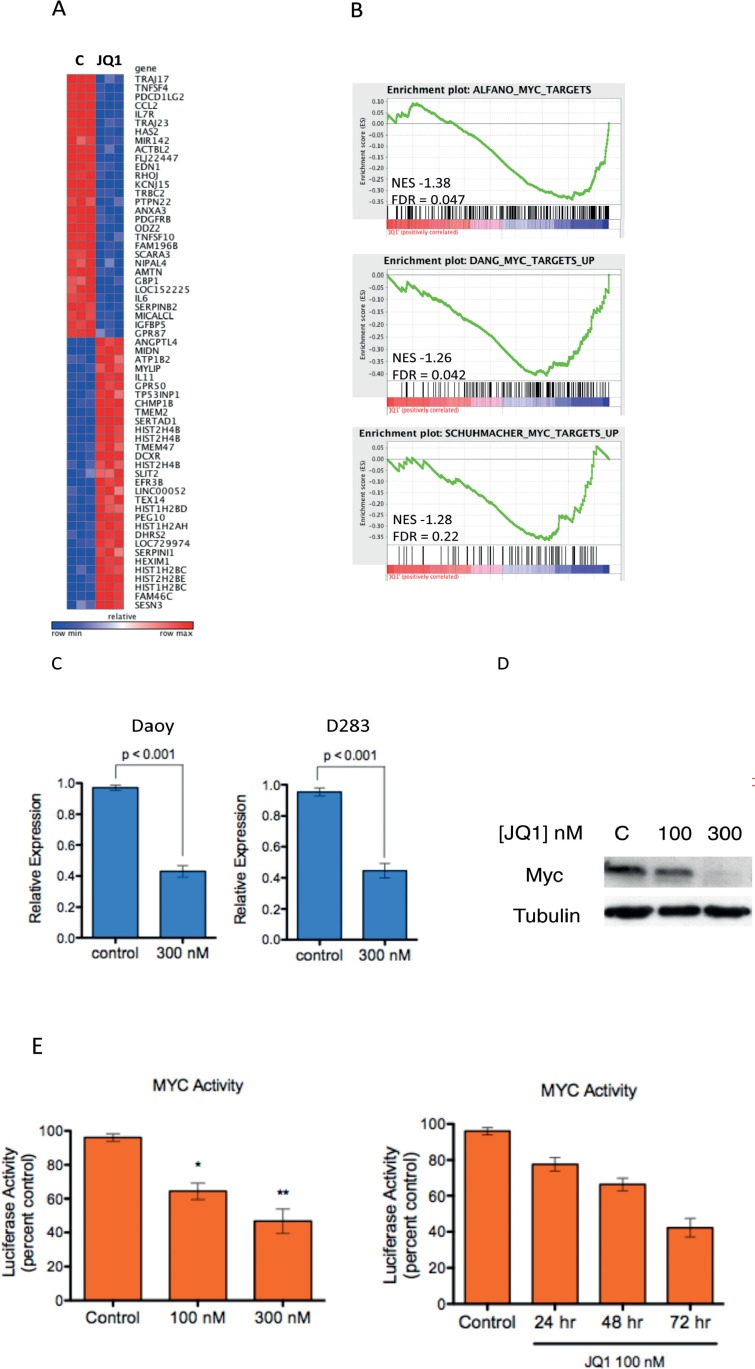
Inhibition of MYC-Dependent Transcription by the Bromodomain Inhibitor JQ1 in medulloblastoma **(A)** Heat map depiction of the top 30 up and down regulated genes (p < 0.01, FDR < 0.05) following JQ1 treatment in Daoy medulloblastoma cells. **(B)** GSEA of three MYC-dependent gene sets (Alfano et al., 2010, Zeller et al., 2003, Schuhmacher et al., 2001) in transcriptional profiles of Daoy medulloblastoma cells treated (red) or untreated (blue) with JQ1. **(C)** Expression of c-MYC mRNA in medulloblastoma cells treated with 300nM JQ1 or control DMSO treated. **(D)** Immunoblot analysis demonstrates that JQ1 down regulates MYC protein expression in Daoy cells. **(E)** A luciferase based reporter assay demonstrates that MYC responsive transcription is inhibited by JQ1 compared to DMSO control treated cells in a concentration and time dependent fashion in Daoy cells.

Recent studies have demonstrated that targeting c-MYC signaling by inhibition of the Bromodomain and extraterminal (BET) Domain 4 (BRD4) is a promising new avenue[16, 28]. Importantly new chemical inhibitors of BRD4 make this approach enticing[21]. Among these inhibitors, JQ1 is the best studied because it has been made freely available by the Bradner laboratory[20]. Because c-MYC is a key driver of high risk medulloblastoma we first asked whether BRD4 inhibition by JQ1 would alter c-MYC driven signaling in medulloblastoma cells. We evaluated the impact of 300nM JQ1 on c-MYC signaling in a well-characterized medulloblastoma cell line (Daoy) using genome wide transcriptional profiling. The top 30 up and down regulated genes are depicted in Figure 1A and the full list is presented in Supplementary Table S1. Low dose (300nM) JQ1 treatment resulted in a significant change in transcription with 1156 transcripts down regulated by JQ1 (p < 0.05,FDR < 0.05 and fold change < 0.67) and 635 transcripts up regulated by JQ1 (p < 0.05,FDR< 0.05 and fold change > 1.5). To more specifically examine the impact of JQ1 on c-MYC driven transcriptional programs we queried the gene expression data with unbiased gene set enrichment analysis (GSEA, reference [27]) using 3 well-validated c-MYC dependent gene signatures publically available from the Molecular Signatures Database (MSigDB, http://www.broadinstitute.org/gsea/msigdb). All three c-MYC related gene sets were statistically enriched among genes down regulated by JQ1 (Figure 1B), suggesting that JQ1 supresses MYC driven genomic programs in medulloblastoma.

Recent studies suggest that the activity of JQ1 is in part due to direct regulation of c-MYC transcription[16]. Therefore we examined the expression of c-MYC in JQ1 treated medulloblastoma cells. Treatment with JQ1 potently decreased c-MYC transcripts (Figure 1C and Supplementary Figure S1A). Immunoblotting analysis confirmed that JQ1 decreased expression of the c-MYC protein in medulloblastoma cells (Figure 1D and Supplementary Figure S1B and C). To further confirm that the JQ1 effects in medulloblastoma cells were a result of suppressing c-MYC we sought to directly evaluate MYC activity. Daoy cells were transfected with control or c-MYC responsive luciferase vectors (SA Bioscience) and treated with JQ1 for 48 hours followed by measurement of luciferase activity as previously described by us[29]. Treatment with JQ1 strongly suppressed luciferase activity in the Daoy cells transfected with c-MYC responsive luciferase vector compared to the DMSO treated control cells in a dose and time dependent manner (Figure 1E). Expression of BRD4 is not increased in medulloblastoma samples compared to normal cerebellum (Supplementary Figure S2) further suggesting the activity of JQ1 is due to BRD4 mediated, c-MYC driven signaling.

Taken together these data provide compelling evidence that JQ1 down regulates c-MYC driven transcription in medulloblastoma cell lines; these data are consistent with activity observed in hematologic malignancies and analogous to that seen in MYCN driven neuroblastoma [16, 24].

### BRD4 inhibition suppresses medulloblastoma cell growth and induces a cell cycle arrest

**Figure 2:**
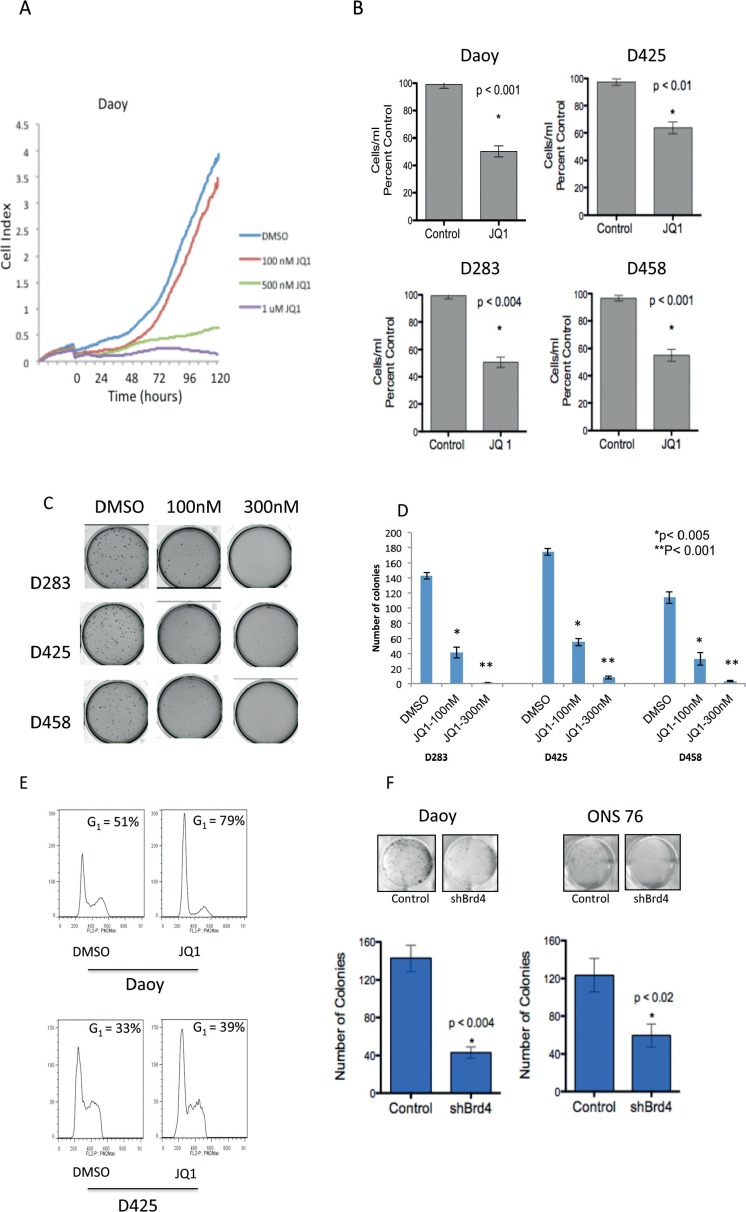
Inhibition of medulloblastoma cell growth by JQ1 *in vitro* **(A)** Growth curves for Daoy cells treated with varying concentrations of JQ1 measured by real time cell analysis., **(B)** Activity of JQ1 (300nM) against a panel of medulloblastoma cell lines measured using the ViaCount assay. **(C)** Methyl cellulose assays showing inhibition of colony formation in the MYC amplified cell lines D283, D425 and D458 by JQ1. **(D)** Quantification of colony formation shown in 2C demonstrating a statistically significant decrease in the number of colonies in JQ1 treated medulloblastoma cells. **(E)** Flow cytometry plots for Daoy and D425 cells show increased G1 cell cycle arrest in JQ1 (300nM) treated cells compared to DMSO control. **(F)** Colony focus assay showing shRNA mediated depletion of BRD4 results in diminished colony formation in Daoy and ONS-76 medulloblastoma cells.

To characterize the phenotypic consequences of BRD4 inhibition on medulloblastoma cells we first determined the impact of JQ1 treatment on medulloblastoma cell growth. JQ1 treatment potently suppressed Daoy medulloblastoma cell growth in a dose dependent manner as evaluated by the RTCA xCELLigence system (ACEA Biotechnology) (Figure 2A). Using a second flow-cytometry based method we confirmed that JQ1 (300nM) effectively inhibits cell growth in a panel of medulloblastoma cell lines (Figure 2B). Moreover treatment with JQ1 significantly attenuated long-term anchorage independent growth of multiple MYC driven medulloblastoma cell lines (p < 0.005, Figure 2C and D). Importantly 3 of the cell lines (D283, D425, D458) are known to possess c-MYC translocations (M. Taylor, personal communication) and all cell lines express c-MYC at significantly higher levels than normal cerebellum (Supplementary Figure S3). In concert with the growth inhibition JQ1 treatment induced a G_1_ cell cycle arrest accompanied by a decrease in the percentage of S-phase cells as shown for Daoy and D425 medulloblastoma cells (Figure 2E) and summarized for all cell lines in Supplementary Table S2.

JQ1 effects in multiple myeloma and AML (acute myeloid leukemia) are associated with inhibition of a particular Bromodomain protein, BRD4[16, 28]. Hence we sought to determine whether genetic inhibition of BRD4 could phenocopy JQ1 treatment. BRD4 was depleted in 2 medulloblastoma cell lines using shRNA (Supplementary Figure S4A). There was a significant decrease in the ability of medulloblastoma cells to form colonies in BRD4 depleted cells (Figure 2F). Furthermore BRD4 depleted cells showed an associated decrease in c-MYC transcript (Supplementary Figure S4B).

### JQ1 treatment suppresses stem cell associated signaling and inhibits medulloblastoma tumor cell self-renewal

**Figure 3:**
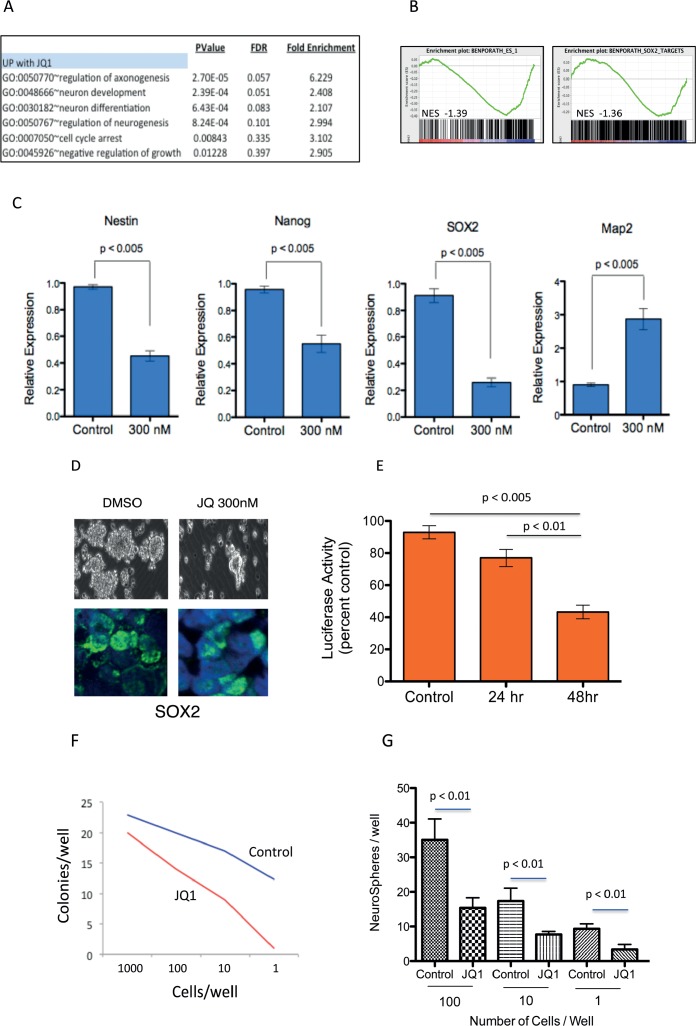
JQ1 suppresses stem cell associated signaling and inhibits medulloblastoma tumor cell self-renewal **(A)** Gene ontology analysis of gene expression from JQ1 treated cells demonstrates induction of differentiation pathways. **(B)** GSEA of ES cell associated gene set and SOX2 dependent gene set in transcriptional profiles of Daoy medulloblastoma cells treated (red) or untreated (blue) with JQ1. **(C)** Expression of stem cell associated markers (Nestin, Nanog, SOX2) and differentiation marker (MAP2) in medulloblastoma cells treated with 300nM JQ1 or control DMSO treated controls. **(D)** Light microscopy and Immunoflurescent images of SOX2 expression in DMSO control or JQ1 treated D283 medulloblastoma cell neurospheres. **(E)** A luciferase based reporter assay demonstrates that SOX2 responsive transcription is inhibited by JQ1 compared to DMSO control treated cells. **(F)** Limiting dilution assay of control (Blue line) or JQ1 (300nM) treated (red line) Daoy cells demonstrating significant inhibition of colony formation by JQ1. **(G)** Limiting dilution assay of control or JQ1 (300nM) treated D283 cells demonstrating significant inhibition of neurosphere formation by JQ1.

Medulloblastoma tumor cells are associated with the ability for augmented self-renewal and limited terminal neuronal differentiation[30]. We therefore examined whether JQ1 influences the differentiation state of medulloblastoma cells. We analyzed the JQ1 modulated gene expression using The **D**atabase for **A**nnotation, **V**isualization and **I**ntegrated **D**iscovery (**DAVID**, http://david.abcc.ncifcrf.gov/)[31]. We found that genes associated with neuronal differentiation processes were poorly expressed in medulloblastoma cells and significantly enriched in transcriptional programs upregulated by JQ1 (Figure 3A). To further investigate the impact of JQ1 on medulloblastoma cell differentiation we performed gene set enrichment analysis of JQ1 treated cells using stem cell associated signatures from MSigDB. GSEA revealed marked down regulation of stem cell associated genes after JQ1 treatment including global ESC (embryonic stem cells) signatures as well as transcriptional programs regulated by SOX2, Nanog and OCT4 (Figure 3B and Supplementary Figure S5). We next examined expression of specific neuronal associated stem cell markers in control versus JQ1 treated cells. Treatment with JQ1 strongly suppressed expression of SOX2, Nestin and Nanog, (Figure 3C) all genes associated with neural stem cells and medulloblastoma[32, 33]. Conversely expression of MAP2, a differentiation maker, in neural stem cells and medulloblastoma was strongly induced by JQ1 (Figure 3C and Supplementary Figure S6). To examine the impact of JQ1 in further detail we treated c-MYC translocated D283 medulloblastoma cells with JQ1 and performed immunofluorescence for SOX2. JQ1 decreased expression of SOX2 protein in medulloblastoma cells grown under serum free neurosphere promoting culture conditions (Figure 3D). Further the activity of SOX2 was significantly attenuated by JQ1 (p < 0.01) as measured by a SOX2 responsive luciferase reporter assay (Figure 3E). To evaluate if JQ1 functionally impacted a stem cell phenotype we next performed an *in vitro* limiting dilution tumor stem cell assay. Daoy cells were grown as neurospheres in serum free conditions for 48 hours and then dissociated and seeded into 96-well plates in a limiting dilution from 1000 cells/well to 1 cell/well. Cells were cultured in serum free conditions for 7 days and colonies counted. The number of neurospheres per well was plotted against the number of cells seeded per well. JQ1 repressed the formation of new neurospheres by Daoy cells indicating a suppression of tumor cell self-renewal (Figure 3F). Similarly D283 formed significantly fewer neurospheres when treated by JQ1 (Figure 3G). Further genetic inhibition of BRD4 with shRNA phenocopied the JQ1 treatment and significantly decreased neurosphere formation of medulloblastoma cells (Supplementary Figure S7).

Together these findings indicate that BRD4 prevents differentiation of medulloblastoma cells by enforcing a stem cell transcriptional program and promoting tumor cell self-renewal.

### JQ1 promotes senescence in medulloblastoma cells

**Figure 4:**
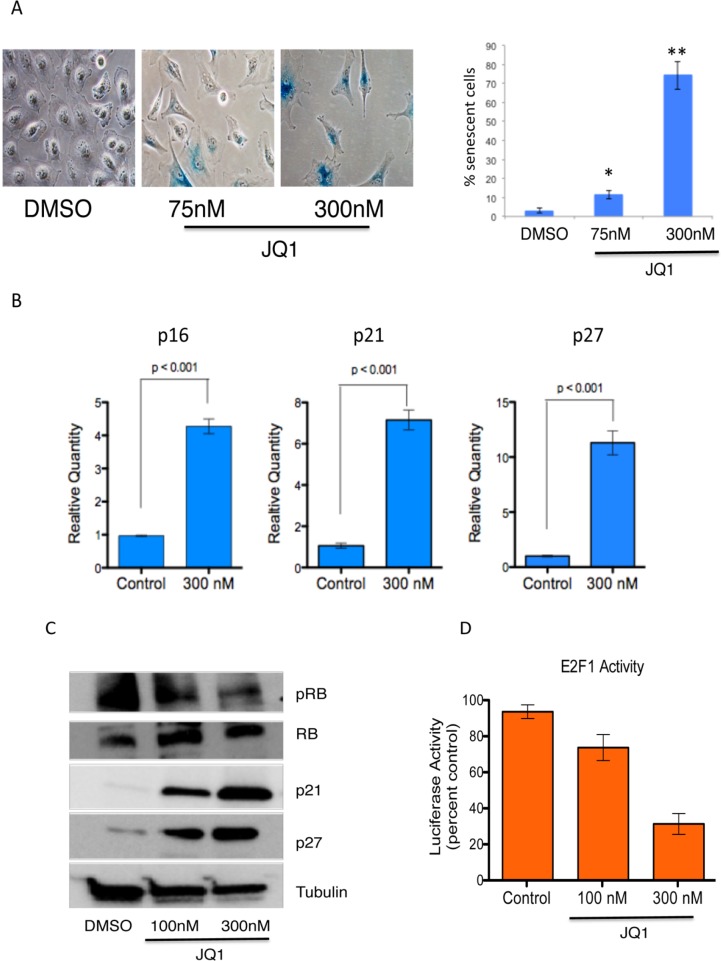
Induction of senescence in medulloblastoma cells by JQ1 **(A)** Representative images of senescence associated β-galactosidase in Daoy cells treated with DMSO and quantification of senescence associated β-galactosidase positive cells per high power field. **(B)** Expression of senescence-associated marker (p16, p21, p27) mRNA in medulloblastoma cells treated with 300nM JQ1 or control DMSO treated controls. **(C)** Immunoblot analysis of senescence associated proteins in JQ1 treated cells. **(D)** A luciferase based reporter assay demonstrates that E2F1 activity is inhibited by JQ1 compared to DMSO control treated cells

To further investigate the mechanism of JQ1 activity in medulloblastoma we asked whether the G_0_–G_1_ arrest we observed was associated with senescence given that tumor cells often undergo senescence upon inhibition of MYC[34]. First we treated Daoy medulloblastoma cells with 75 or 300 nM JQ1 and measured activity of senescence associated β-galactosidase after 7 days. JQ1 strongly induced senescence- β-galactosidase staining (Figure 4A) indicating increased senescence. To confirm these data we measured expression of cell cycle related genes that are known to be associated with c-MYC inactivation associated senescence[34]. JQ1 increased expression of p16, p21 and p27 transcripts in medulloblastoma cells (Figure 4B). Western blot analysis further revealed potent induction of p21 and p27 with concomitant decrease in phosphorylated RB protein (Figure 4C). Because MYC associated G1 cell cycle transitions are associated with activity of Rb bound E2F1 we further measured activity of E2F1. MYC drives E2F1 activity by promoting phosphorylation and degradation of RB[11]. Treatment of medulloblastoma cells with JQ1 potently suppressed E2F1 activity compared to control treated cells as measured by a E2F1 responsive luciferase reporter (Figure 4D).

These data further establish MYC inactivation associated senescence as a key mechanism of BRD4 inhibition mediated suppression of medulloblastoma cell growth.

### BRD4 inhibition displays anti-proliferative effects in a medulloblastoma model *in vivo*

**Figure 5:**
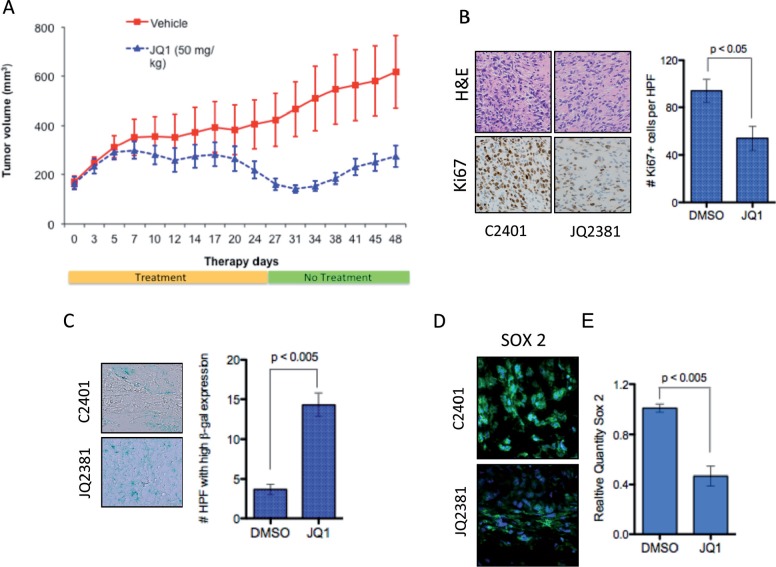
*In vivo* activity of JQ1 in medulloblastoma xenografts **(A)** Tumor volume of medulloblastoma cell xenografts in DMSO control (Red) or JQ1(blue) treated immunocompromised mice. **(B)** Representative images of H&E staining and Ki67 IHC in control treated tumor (C2401) or JQ1 treated tumor (JQ2381). **(C)** Representative images and quantification of senescence associated β-galactosidase in control treated tumor (C2401) or JQ1 treated tumor (JQ2381). **(D)** Immunofluoresence of SOX2 protien in in control treated tumor (C2401) or JQ1 treated tumor (JQ2381). **(E)** Expression of SOX2 mRNA in control or JQ1 treated xenograft tumors (n = 3 each).

Based on our *in vitro* data we next evaluated the therapeutic application of JQ1 *in vivo*. Daoy medulloblastoma cells were grown s.c. in immunocompromised mice until tumors were apparent (~150mm^2^). JQ1 was administered at 50mg/kg i.p. 5 days a week for 4 weeks and tumor size monitored for a further 20 days. Daoy tumors grew significantly slower in JQ1 treated mice compared to DMSO treated controls (Figure 5A). Importantly this dosing scheme resulted in very little toxicity with no significant change in animal weights over the study period (Supplementary Figure S8). To further evaluate *in vivo* activity of JQ1, a cohort of animals were sacrificed after a 5-day treatment and tumors assessed for proliferation. JQ1 potently inhibited proliferation as demonstrated by decreased Ki67 staining compared to controls and strongly induced senescence in treated tumors (Figure 5B & C and Supplementary Figure S9). Excitingly JQ1 was effective in depleting SOX2 positive tumor cells *in vivo* (Figure 5D) and in decreasing SOX2 mRNA expression (Figure 5E) further confirming that BRD4 inhibition suppresses the tumor stem cell phenotype in medulloblastoma. These data demonstrate the significant *in vivo* antitumor activity of JQ1 and establish a potential therapeutic strategy for BET inhibition in medulloblastoma.

### JQ1 diminishes tumor cell proliferation of patient derived medulloblastoma *ex-vivo*

**Figure 6:**
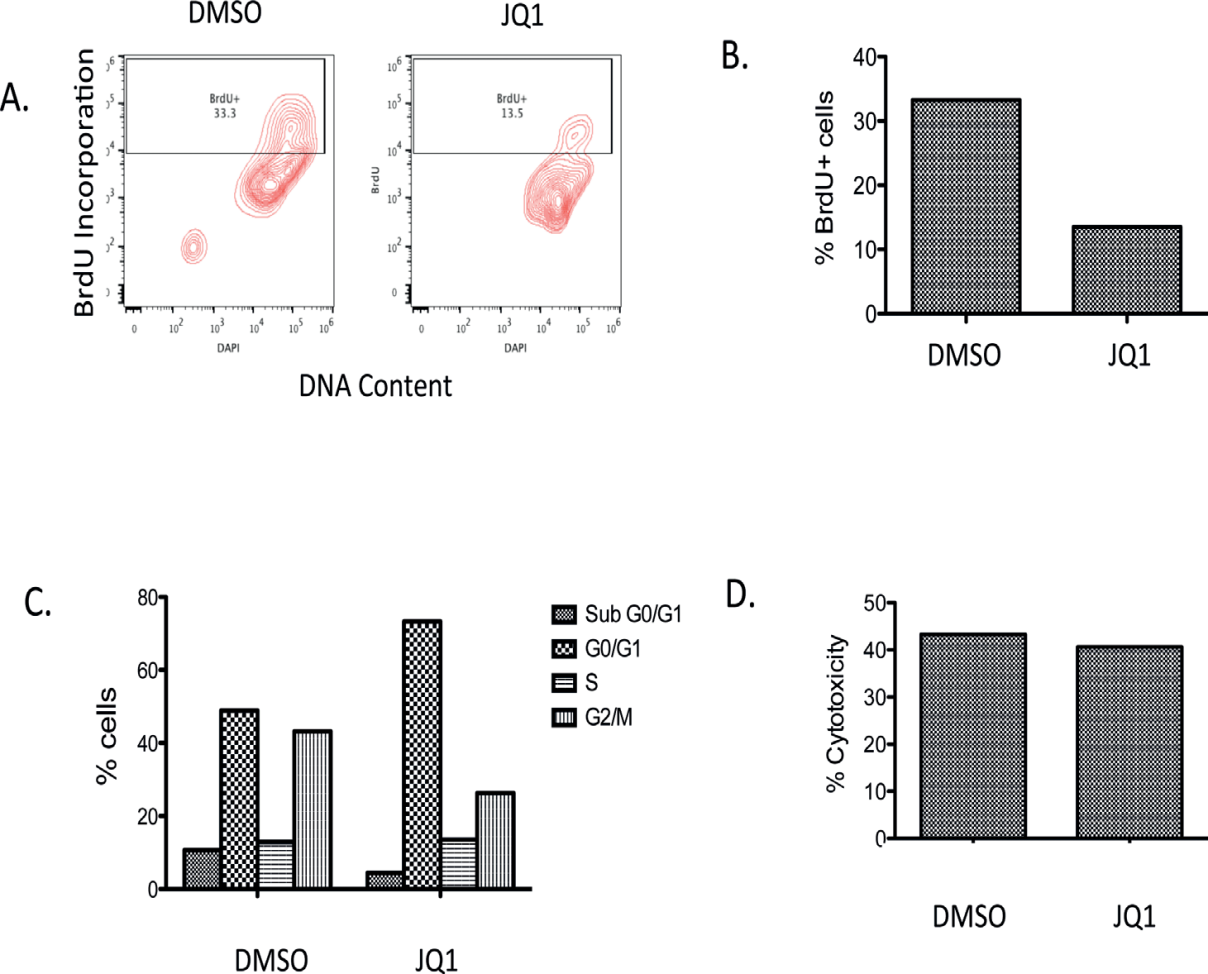
Analysis of BrdU incorporation, cell viability and LDH activity in a primary medulloblastoma patient tumor slice culture with JQ1 (300nM) treatment or DMSO vehicle control **(A)** Representative Bivariate distribution of cellular DNA content and BrdU incorporation with JQ1 treatment **(B)** Quantitative analysis of BrdU incorporation in cells with JQ1. **(C)** Distribution of cells in the cell cycle phase. **(D)** Cytotoxicity measurement of JQ1 from LDH assay.

We next evaluated the activity of JQ1 in a slice culture model of primary medulloblastoma. This model allows tumor to grow intact with all the supporting cells and microenvironment *ex vivo*. Tumor harvested from a medulloblastoma patient was placed in cell culture *en block* at time of surgery. Tissue was then sliced in to 0.33cm thick sections, placed on cell growth inserts and cultured in slice culture medium. Five days after initiation of the slice culture, DMSO or 300nM JQ1 was added to media and sections cultured for a further 7 days. BrdU was then added to sections and tissues incubated for a further 2 days. Tissues were then collected and evaluated for BrdU incorporation, cell cycle analysis and cytotoxicity assays. JQ1 treated slice cultures incorporated less BrdU compared to DMSO (Figure 6A and B). Further JQ1 induced a significant G1 cell cycle arrest in the primary medulloblastoma cell culture (Figure 6C). However JQ1 did not induce appreciable cytotoxicity in the slice culture model. These data are consistent with our *in vitro* and *in vivo* studies and further emphasize the utility of JQ1 in medulloblastoma.

### Transcriptional programs suppressed by JQ1 are associated with adverse risk in medulloblastoma patients

**Figure 7:**
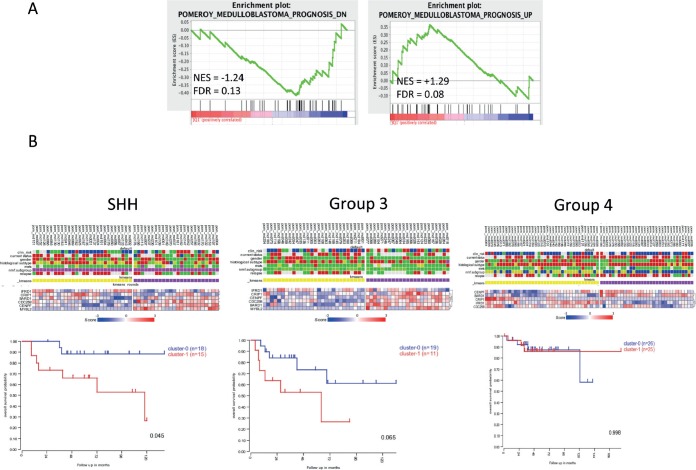
Clinical implication of JQ1 suppressed transcriptional programs in medulloblastoma patients **(A)** GSEA of an adverse risk gene set and a good prognosis gene set in transcriptional profiles of Daoy medulloblastoma cells treated (red) or untreated (blue) with JQ1. **(B)** *k*-means clustering and Kaplan-Meir analysis of a 6 gene signature by medulloblastoma genomic subgroups in a series of 199 primary medulloblastoma samples.

We next sought to determine the clinical relevance of JQ1 mediated changes in transcriptional programs. Early transcriptomic studies in medulloblastoma identified gene signatures that are associated with adverse or improved outcomes in medulloblastoma[35]. Using these signatures we performed GSEA on the transcriptomic changes in JQ1 treated medulloblastoma cells. Genes identified by Pomeroy *et al*, as adverse markers of medulloblastoma outcomes (Pomeroy_Medulloblastoma_Prognosis_Down) were significantly suppressed by JQ1 treatment of Daoy cells (NES = −1.24, FDR = 0.13, Figure 7A). Conversely genes associated with improved outcomes in medulloblastoma (Pomeroy_Medulloblastoma_Prognosis_Up) were significantly enriched in the genomic program up-regulated by JQ1 (NES = +1.29, FDR = 0.08) as shown in Figure 7A. We next performed leading edge analysis using MSigDB to identify the genes specifically contributing to the enrichment score. Using this method we identified a set of 6 genes that contributed most to the Pomeroy adverse gene set being negatively enriched in our JQ1 treated gene signatures (list in Supplementary Figure S10A). These 6 genes are significantly suppressed by JQ1 in medulloblastoma cells and were identified as part of a larger gene set that is highly predictive of adverse outcomes[35]. To further probe the clinical relevance of this gene signature we conducted *k*-means clustering of the 6 genes using R2 (R2: microarray analysis and visualization platform; http://r2.amc.nl) in a series of 199 primary medulloblastoma samples[5]. This clustering analysis yielded separation of the 199-medulloblastoma tumors in to 2 clear groups (Supplementary Figure S10B). Kaplan-Meir survival analysis of these two groups did not reveal any differences in outcomes in medulloblastoma samples as a whole (Supplementary Figure S10C). However when we examined the six-gene signature on a subgroup basis we found that that this six-gene set was predictive of outcomes in SHH (p = 0.045) and suggestive of outcome prediction in Group3 tumors (p =0.065) but not associated with outcomes in Group 4 tumors (Figure 7B). Not enough Wnt signaling tumors were available for this analysis. These data suggest that BRD4 inhibition is a promising strategy for specific subgroups of medulloblastoma patients.

## DISCUSSION

Medulloblastoma is a clinically and genomically heterogeneous disease that is frequently treated with a “one size fits all” approach using surgery, radiation and chemotherapy[7]. Unfortunately this approach results in very poor outcomes for MYC driven tumors [8]. Here we show that the BET domain protein BRD4 is a mediator of medulloblastoma growth in the context of MYC pathway activation. Chemical inhibition of BRD4 with JQ1 was sufficient to inhibit growth of medulloblastoma cells *in vitro* and *in vivo*. Moreover JQ1 suppressed genomic programs of stem cell signaling and suppressed tumor cell self-renewal. JQ1 induced a potent senescence phenotype associated with induction of cell cycle inhibitory proteins. These data suggest that BRD4 inhibition is a potential therapeutic target in medulloblastoma.

BRD4 is a member of the BET family of transcription regulators that function as epigenetic readers[36]. BRD4 has two bromodomains, which bind to acetylated lysine residues in histone H3 and H4[37]. After binding to acetylated chromatin, BRD4 regulates transcription by recruiting chromatin modifiers, nucleosome remodeling complexes, and transcriptional co-activators[18]. BRD4 is also required for maintaining cell-cycle progression and BRD4 knockout in murine models is embryonic lethal [36]. BRD4's role in cancer was initially appreciated when the presence of a fusion protein was identified in a very aggressive form of squamous cell carcinoma[38]. Since then BRD4 has been implicated in multiple myeloma as well as MYC driven tumors such as AML and neuroblastoma. Importantly these tumors types were responsive to BRD4 inhibition by JQ1, a small molecule inhibitor of BRD4. JQ1 binds to the bromodomain and displaces BRD4 from acetylated lysines on chromatin[20]. Pursuing the rational that JQ1 would broadly affect other MYC driven tumors we tested this hypothesis on a panel of medulloblastoma cells. The JQ1 altered transcriptomic profile of medulloblastoma cells was heavily enriched for c-MYC-dependent gene signatures consistent with recent reports in multiple myeloma and AML [16, 28]. Interestingly a recent report also demonstrates that JQ1 alters MYCN driven transcription in neuroblastoma suggesting the potential for targeting of multiple MYC family members with JQ1[24].

We observed that JQ1 strongly curbed stem cell associated genomic programs. Our data are consistent with the concept that a MYC centered transcriptomic program is critical for embryonic stem cell maintenance and also accounts for the similarities between embryonic stem cell cell and cancer cell transcriptional programs[39]. We confirmed the alterations in stem cell associated genomic programs by showing that expression and activity of critical stem cell genes, SOX2 and Nestin, were attenuated by JQ1. SOX2 is a well-known marker of highly pluripotent stem cells in the CNS[32]. Nestin is a marker for Neural stem cells (NSC) and its expression is lost as NSCs differentiate into lineage-restricted neuronal and glial progenitors[32]. Recently SOX2 was also directly implicated in medulloblastoma[40]. Ahlfeld *et al* demonstrated that SHH-associated medulloblastoma could be initiated from SOX2-positive cerebellar granule cells (CGNP) and ablation of SOX2 in CGNP resulted in significantly diminished capacity for proliferation[40]. Previous reports have also implicated the role of Nestin-expressing progenitors in medulloblastoma initiation[41]. Moreover a recent report demonstrates that a rare population of Nestin-expressing progenitors (NEPs) reside in the cerebellum and exhibit decreased expression of DNA repair genes; upon aberrant activation of SHH signaling these NEP give rise to medulloblastoma tumors[42]. Further, multiple reports have demonstrated that tumor stem cells exist in medulloblastoma and are associated with resistance to conventional therapy[30, 43, 44]. We showed that JQ1 inhibited medulloblastoma tumor cell self-renewal and promoted a differentiation phenotype. These findings are analogous to the hematopoietic system where BRD4 inhibition promoted a macrophage like differentiation of AML cells and depleted the leukemia stem cell compartment [28, 45]. Together these data suggest that BRD4 inhibition can be a highly effective strategy to target tumor stem cells in medulloblastoma and other MYC driven tumors.

In the context of decreased expression of stem cell markers we found that JQ1 mediated BRD4 inhibition drives senescence in medulloblastoma cells with a significant up regulation of cell cycle kinase inhibitors. These data further highlight the role of MYC in cellular senescence. Wu *et al* previously showed that cellular senescence is a key mechanism of sustained tumor regression in MYC driven tumors[34]. They demonstrated that genetic suppression of MYC in MYC-induced lymphoma resulted in increased senescence associated acidic **β** - gal staining and heterochromatin formation[34]. Recently using an inducible c-MYC expression construct Pei *et al* demonstrated that c-MYC was required for both initiation and maintenance of tumors in a murine model of MYC driven medulloblastoma. [46]. Similarly Swartling *et al* demonstrated that MYCN was required for both initiation and maintenance of medulloblastoma tumors in an inducible murine model[47]. Together these studies suggest that chronic inhibition of MYC with low dose BET domain inhibitors may be the optimal approach to treating these highly aggressive tumors.

JQ1 suppresses tumor growth of several tumor types including AML and neuroblastoma *in vivo[24, 28]*. However JQ1 is a first generation chemical inhibitor of BRD4 with an *in vivo* half-life of only one hour in rodent models[20]. Work is underway to optimize JQ1. In addition several new BET inhibitors have been described and are under clinical development[48]. Remarkably our data show that JQ1 can suppress growth of established medulloblastoma tumors in mouse xenografts. The next step will be to evaluate patient derived xenografts in an orthotopic model. Given that JQ1 has excellent CNS penetration this approach should be feasible[20]. Moreover it will be interesting to evaluate BET inhibition in the context of a minimal residual disease model, which is the most likely clinical scenario in which such inhibitors will be used. Likewise it will be important to examine whether BET inhibition can co-operate with current therapeutic agents to further increase efficacy of treating medulloblastoma. For example BET inhibition may radio-sensitize medulloblastoma cells in light of JQ1 activity at the G1-S cell cycle boundary. Certainly recent reports implicating BRD4 in DNA damage signaling that is independent of MYC activity suggests such a strategy would be feasible[49]. Combining BET inhibition with HDAC inhibition is another promising avenue in medulloblastoma. HDAC inhibition is known to inhibit medulloblastoma cell growth and induce expression of tumor suppressor genes[50, 51]. Targeting both erasers and readers of acetyl histone modifications could modulate transcriptional programs to drive medulloblastoma cells towards a differentiated state.

Of note we identified a six-gene signature that is associated with adverse prognosis in medulloblastoma and whose expression is repressed by JQ1. Importantly this signature identifies a subset of patients with adverse outcomes in both SHH and Group 3 tumors but not in Group 4 tumors. These findings highlight the diverse role of MYC in a sub group context. It is no surprise that Group 3 tumors are associated with this signature given that c-MYC amplification is a major genetic hallmark of this particular subtype of medulloblastoma. Interestingly SHH tumors are known to have increased expression and amplification of MYCN, which may explain why SHH tumors also enrich with our 6-gene signature. In addition one or more of the proteins coded by the six-gene signature could represent a viable biomarker for identifying medulloblastoma patients likely to respond to BET domain inhibition.

In summary we establish the concept that MYC driven medulloblastoma can be targeted with BET domain inhibition and demonstrate the feasibility of this approach *in vivo*. Excitingly, while this work was in preparation two other reports demonstrated that BRD4 inhibition with JQ1 can inhibit medulloblastoma cell growth in vitro and in vivo[25, 26]. Henssen *et al* showed that JQ1 reduces cell viability and proliferation and induces apoptosis in human medulloblastoma cell lines *in vitro* and *in vivo*[25]. Bandopadhayay *et al* also showed that JQ1 reduced cell proliferation and induced apoptosis in MYC-amplified medulloblastoma *in vitro* and prolonged survival in xenograft models[26]. Our data extend these findings by demonstrating that BET inhibition targets tumor stem cells and suppresses a genomic signature associated with adverse outcomes in medulloblastoma. We are now pursuing further studies to more clearly understand the role of BRD4 function in medulloblastoma and the application of BRD4 inhibition in clinically relevant models.

## SUPPLEMENTARY FIGURES AND TABLES



## References

[1] Dhall G (2009). Medulloblastoma. J Child Neurol.

[2] Mulhern RK, Palmer SL, Merchant TE, Wallace D, Kocak M, Brouwers P, Krull K, Chintagumpala M, Stargatt R, Ashley DM, Tyc VL, Kun L, Boyett J, Gajjar A (2005). Neurocognitive consequences of risk-adapted therapy for childhood medulloblastoma. J Clin Oncol.

[3] Thompson MC, Fuller C, Hogg TL, Dalton J, Finkelstein D, Lau CC, Chintagumpala M, Adesina A, Ashley DM, Kellie SJ, Taylor MD, Curran T, Gajjar A, Gilbertson RJ (2006). Genomics identifies medulloblastoma subgroups that are enriched for specific genetic alterations. J Clin Oncol.

[4] Kool M, Koster J, Bunt J, Hasselt NE, Lakeman A, van Sluis P, Troost D, Meeteren NS, Caron HN, Cloos J, Mrsic A, Ylstra B, Grajkowska W, Hartmann W, Pietsch T, Ellison D (2008). Integrated genomics identifies five medulloblastoma subtypes with distinct genetic profiles, pathway signatures and clinicopathological features. PloS one.

[5] Cho YJ, Tsherniak A, Tamayo P, Santagata S, Ligon A, Greulich H, Berhoukim R, Amani V, Goumnerova L, Eberhart CG, Lau CC, Olson JM, Gilbertson RJ, Gajjar A, Delattre O, Kool M (2012). Integrative genomic analysis of medulloblastoma identifies a molecular subgroup that drives poor clinical outcome. J Clin Oncol.

[6] Northcott PA, Korshunov A, Witt H, Hielscher T, Eberhart CG, Mack S, Bouffet E, Clifford SC, Hawkins CE, French P, Rutka JT, Pfister S, Taylor MD (2011). Medulloblastoma comprises four distinct molecular variants. J Clin Oncol.

[7] Northcott PA, Korshunov A, Pfister SM, Taylor MD (2012). The clinical implications of medulloblastoma subgroups. Nat Rev Neurol.

[8] Kool M, Korshunov A, Remke M, Jones DT, Schlanstein M, Northcott PA, Cho YJ, Koster J, Schouten-van Meeteren A, van Vuurden D, Clifford SC, Pietsch T, von Bueren AO, Rutkowski S, McCabe M, Collins VP (2012). Molecular subgroups of medulloblastoma: an international meta-analysis of transcriptome, genetic aberrations, and clinical data of WNT, SHH, Group 3, and Group 4 medulloblastomas. Acta neuropathologica.

[9] Ellison DW, Kocak M, Dalton J, Megahed H, Lusher ME, Ryan SL, Zhao W, Nicholson SL, Taylor RE, Bailey S, Clifford SC (2011). Definition of disease-risk stratification groups in childhood medulloblastoma using combined clinical, pathologic, and molecular variables. J Clin Oncol.

[10] Ryan SL, Schwalbe EC, Cole M, Lu Y, Lusher ME, Megahed H, O'Toole K, Nicholson SL, Bognar L, Garami M, Hauser P, Korshunov A, Pfister SM, Williamson D, Taylor RE, Ellison DW (2012). MYC family amplification and clinical risk-factors interact to predict an extremely poor prognosis in childhood medulloblastoma. Acta neuropathologica.

[11] Swartling FJ (2012). Myc proteins in brain tumor development and maintenance. Upsala journal of medical sciences.

[12] Northcott PA, Shih DJ, Peacock J, Garzia L, Morrissy AS, Zichner T, Stutz AM, Korshunov A, Reimand J, Schumacher SE, Beroukhim R, Ellison DW, Marshall CR, Lionel AC, Mack S, Dubuc A (2012). Subgroup-specific structural variation across 1,000 medulloblastoma genomes. Nature.

[13] Felsher DW, Bishop JM (1999). Reversible tumorigenesis by MYC in hematopoietic lineages. Molecular cell.

[14] Fukazawa T, Maeda Y, Matsuoka J, Yamatsuji T, Shigemitsu K, Morita I, Faiola F, Durbin ML, Soucek L, Naomoto Y (2010). Inhibition of Myc effectively targets KRAS mutation-positive lung cancer expressing high levels of Myc. Anticancer research.

[15] Shalaby T, von Bueren AO, Hurlimann ML, Fiaschetti G, Castelletti D, Masayuki T, Nagasawa K, Arcaro A, Jelesarov I, Shin-ya K, Grotzer M (2010). Disabling c-Myc in childhood medulloblastoma and atypical teratoid/rhabdoid tumor cells by the potent G-quadruplex interactive agent S2T1-6OTD. Molecular cancer therapeutics.

[16] Delmore JE, Issa GC, Lemieux ME, Rahl PB, Shi J, Jacobs HM, Kastritis E, Gilpatrick T, Paranal RM, Qi J, Chesi M, Schinzel AC, McKeown MR, Heffernan TP, Vakoc CR, Bergsagel PL (2011). BET bromodomain inhibition as a therapeutic strategy to target c-Myc. Cell.

[17] Mertz JA, Conery AR, Bryant BM, Sandy P, Balasubramanian S, Mele DA, Bergeron L, Sims RJ (2011). Targeting MYC dependence in cancer by inhibiting BET bromodomains. Proceedings of the National Academy of Sciences of the United States of America.

[18] Wu SY, Chiang CM (2007). The double bromodomain-containing chromatin adaptor Brd4 and transcriptional regulation. The Journal of biological chemistry.

[19] Chung CW, Coste H, White JH, Mirguet O, Wilde J, Gosmini RL, Delves C, Magny SM, Woodward R, Hughes SA, Boursier EV, Flynn H, Bouillot AM, Bamborough P, Brusq JM, Gellibert FJ (2011). Discovery and characterization of small molecule inhibitors of the BET family bromodomains. Journal of medicinal chemistry.

[20] Filippakopoulos P, Qi J, Picaud S, Shen Y, Smith WB, Fedorov O, Morse EM, Keates T, Hickman TT, Felletar I, Philpott M, Munro S, McKeown MR, Wang Y, Christie AL, West N (2010). Selective inhibition of BET bromodomains. Nature.

[21] Chung CW (2012). Small molecule bromodomain inhibitors: extending the druggable genome. Progress in medicinal chemistry.

[22] Grayson AR, Walsh EM, Cameron MJ, Godec J, Ashworth T, Ambrose JM, Aserlind AB, Wang H, Evan GI, Kluk MJ, Bradner JE, Aster JC, French CA (2013). MYC, a downstream target of BRD-NUT, is necessary and sufficient for the blockade of differentiation in NUT midline carcinoma. Oncogene.

[23] Ott CJ, Kopp N, Bird L, Paranal RM, Qi J, Bowman T, Rodig SJ, Kung AL, Bradner JE, Weinstock DM (2012). BET bromodomain inhibition targets both c-Myc and IL7R in high-risk acute lymphoblastic leukemia. Blood.

[24] Puissant A, Frumm SM, Alexe G, Bassil CF, Qi J, Chanthery YH, Nekritz EA, Zeid R, Gustafson WC, Greninger P, Garnett MJ, McDermott U, Benes CH, Kung AL, Weiss WA, Bradner JE (2013). Targeting MYCN in neuroblastoma by BET bromodomain inhibition. Cancer discovery.

[25] Henssen A, Thor T, Odersky A, Heukamp L, El-Hindy N, Beckers A, Speleman F, Althoff K, Schafers S, Schramm A, Sure U, Fleischhack G, Eggert A, Schulte JH (2013). BET bromodomain protein inhibition is a therapeutic option for medulloblastoma. Oncotarget.

[26] Bandopadhayay P, Bergthold G, Nguyen B, Schubert S, Gholamin S, Tang Y, Bolin S, Schumacher SE, Zeid R, Masoud S, Yu F, Vue N, Gibson WJ, Paolella BR, Mitra SS, Cheshier SH (2014). BET Bromodomain Inhibition of MYC-Amplified Medulloblastoma. Clinical cancer research : an official journal of the American Association for Cancer Research.

[27] Subramanian A, Tamayo P, Mootha VK, Mukherjee S, Ebert BL, Gillette MA, Paulovich A, Pomeroy SL, Golub TR, Lander ES, Mesirov JP (2005). Gene set enrichment analysis: a knowledge-based approach for interpreting genome-wide expression profiles. Proceedings of the National Academy of Sciences of the United States of America.

[28] Zuber J, Shi J, Wang E, Rappaport AR, Herrmann H, Sison EA, Magoon D, Qi J, Blatt K, Wunderlich M, Taylor MJ, Johns C, Chicas A, Mulloy JC, Kogan SC, Brown P (2011). RNAi screen identifies Brd4 as a therapeutic target in acute myeloid leukaemia. Nature.

[29] Alimova I, Birks DK, Harris PS, Knipstein JA, Venkataraman S, Marquez VE, Foreman NK, Vibhakar R (2013). Inhibition of EZH2 suppresses self-renewal and induces radiation sensitivity in atypical rhabdoid teratoid tumor cells. Neuro Oncol.

[30] Manoranjan B, Venugopal C, McFarlane N, Doble BW, Dunn SE, Scheinemann K, Singh SK (2012). Medulloblastoma stem cells: where development and cancer cross pathways. Pediatric research.

[31] Huang da W, Sherman BT, Lempicki RA (2009). Systematic and integrative analysis of large gene lists using DAVID bioinformatics resources. Nature protocols.

[32] Swartling FJ, Bolin S, Phillips JJ, Persson AI (2013). Signals that regulate the oncogenic fate of neural stem cells and progenitors. Experimental neurology.

[33] Wechsler-Reya R, Scott MP (2001). The developmental biology of brain tumors. Annu Rev Neurosci.

[34] Wu CH, van Riggelen J, Yetil A, Fan AC, Bachireddy P, Felsher DW (2007). Cellular senescence is an important mechanism of tumor regression upon c-Myc inactivation. Proceedings of the National Academy of Sciences of the United States of America.

[35] Pomeroy SL, Tamayo P, Gaasenbeek M, Sturla LM, Angelo M, McLaughlin ME, Kim JY, Goumnerova LC, Black PM, Lau C, Allen JC, Zagzag D, Olson JM, Curran T, Wetmore C, Biegel JA (2002). Prediction of central nervous system embryonal tumour outcome based on gene expression. Nature.

[36] Belkina AC, Denis GV (2012). BET domain co-regulators in obesity, inflammation and cancer. Nature reviews Cancer.

[37] Filippakopoulos P, Picaud S, Mangos M, Keates T, Lambert JP, Barsyte-Lovejoy D, Felletar I, Volkmer R, Muller S, Pawson T, Gingras AC, Arrowsmith CH, Knapp S (2012). Histone recognition and large-scale structural analysis of the human bromodomain family. Cell.

[38] French CA, Ramirez CL, Kolmakova J, Hickman TT, Cameron MJ, Thyne ME, Kutok JL, Toretsky JA, Tadavarthy AK, Kees UR, Fletcher JA, Aster JC (2008). BRD-NUT oncoproteins: a family of closely related nuclear proteins that block epithelial differentiation and maintain the growth of carcinoma cells. Oncogene.

[39] Kim J, Woo AJ, Chu J, Snow JW, Fujiwara Y, Kim CG, Cantor AB, Orkin SH (2010). A Myc network accounts for similarities between embryonic stem and cancer cell transcription programs. Cell.

[40] Ahlfeld J, Favaro R, Pagella P, Kretzschmar HA, Nicolis S, Schuller U (2013). Sox2 requirement in sonic hedgehog-associated medulloblastoma. Cancer research.

[41] Rao G, Pedone CA, Del Valle L, Reiss K, Holland EC, Fults DW (2004). Sonic hedgehog and insulin-like growth factor signaling synergize to induce medulloblastoma formation from nestin-expressing neural progenitors in mice. Oncogene.

[42] Li P, Du F, Yuelling LW, Lin T, Muradimova RE, Tricarico R, Wang J, Enikolopov G, Bellacosa A, Wechsler-Reya RJ, Yang ZJ (2013). A population of Nestin-expressing progenitors in the cerebellum exhibits increased tumorigenicity. Nature neuroscience.

[43] Singh SK, Hawkins C, Clarke ID, Squire JA, Bayani J, Hide T, Henkelman RM, Cusimano MD, Dirks PB (2004). Identification of human brain tumour initiating cells. Nature.

[44] Sun L, Moritake T, Zheng YW, Suzuki K, Gerelchuluun A, Hong Z, Zenkoh J, Taniguchi H, Tsuboi K (2013). In vitro stemness characterization of radio-resistant clones isolated from a medulloblastoma cell line ONS-76. J Radiat Res.

[45] Herrmann H, Blatt K, Shi J, Gleixner KV, Cerny-Reiterer S, Mullauer L, Vakoc CR, Sperr WR, Horny HP, Bradner JE, Zuber J, Valent P (2012). Small-molecule inhibition of BRD4 as a new potent approach to eliminate leukemic stem- and progenitor cells in acute myeloid leukemia AML. Oncotarget.

[46] Pei Y, Moore CE, Wang J, Tewari AK, Eroshkin A, Cho YJ, Witt H, Korshunov A, Read TA, Sun JL, Schmitt EM, Miller CR, Buckley AF, McLendon RE, Westbrook TF, Northcott PA (2012). An animal model of MYC-driven medulloblastoma. Cancer cell.

[47] Swartling FJ, Grimmer MR, Hackett CS, Northcott PA, Fan QW, Goldenberg DD, Lau J, Masic S, Nguyen K, Yakovenko S, Zhe XN, Gilmer HC, Collins R, Nagaoka M, Phillips JJ, Jenkins RB (2010). Pleiotropic role for MYCN in medulloblastoma. Genes & development.

[48] Mirguet O, Gosmini R, Toum J, Clement CA, Barnathan M, Brusq JM, Mordaunt JE, Grimes RM, Crowe M, Pineau O, Ajakane M, Daugan A, Jeffrey P, Cutler L, Haynes AC, Smithers NN (2013). Discovery of Epigenetic Regulator I-BET762: Lead Optimization to Afford a Clinical Candidate Inhibitor of the BET Bromodomains. Journal of medicinal chemistry.

[49] Floyd SR, Pacold ME, Huang Q, Clarke SM, Lam FC, Cannell IG, Bryson BD, Rameseder J, Lee MJ, Blake EJ, Fydrych A, Ho R, Greenberger BA, Chen GC, Maffa A, Del Rosario AM (2013). The bromodomain protein Brd4 insulates chromatin from DNA damage signalling. Nature.

[50] Vibhakar R, Foltz G, Yoon JG, Field L, Lee H, Ryu GY, Pierson J, Davidson B, Madan A (2007). Dickkopf-1 is an epigenetically silenced candidate tumor suppressor gene in medulloblastoma. Neuro Oncol.

[51] Wegener D, Deubzer HE, Oehme I, Milde T, Hildmann C, Schwienhorst A, Witt O (2008). HKI 46F08, a novel potent histone deacetylase inhibitor, exhibits antitumoral activity against embryonic childhood cancer cells. Anti-cancer drugs.

