# Supplementary material for: Inhibition of BRD4 attenuates tumor cell self-renewal and suppresses stem cell signaling in MYC driven medulloblastoma
Source: Oncotarget. 2014 Mar 31;5(9):2355–71. doi: 10.18632/oncotarget.1659 (PMC4058011; doi:10.18632/oncotarget.1659)
Supplement: Supplementary file 1 [file oncotarget-05-2355-s001.pdf]

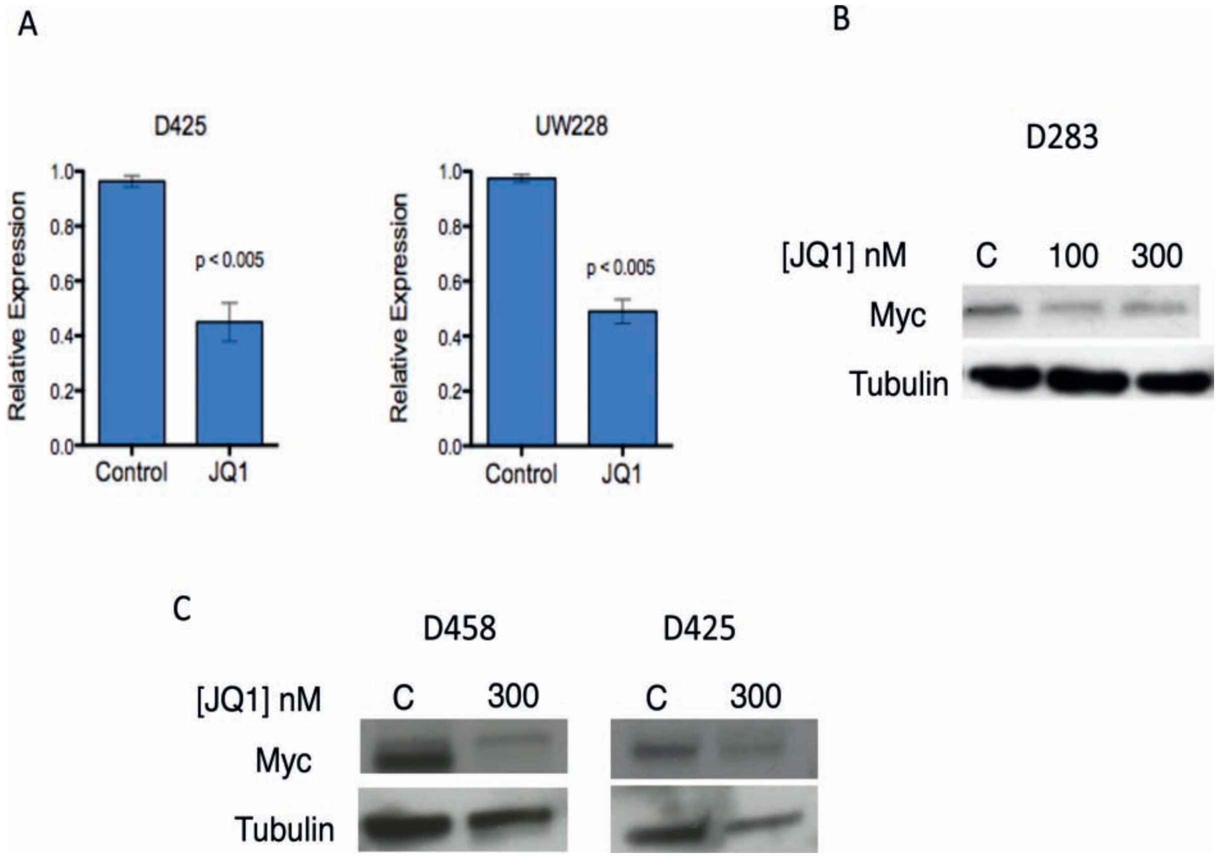

Supplementary Figure S1: Inhibition of MYC Expression by JQ1 in medulloblastoma. (A) Expression of c-MYC mRNA in D425 and UW228 medulloblastoma cells treated with 300nM JQ1 or control DMSO treated controls. **(B)** Immunoblot analysis of MYC protein in D283 cells treated with JQ1 confirms results seen in DAOY cells. **(C)** Immunoblot analysis of MYC protein in D458 and D425 cells treated with JQ1 confirms results seen in DAOY and D283 cells.

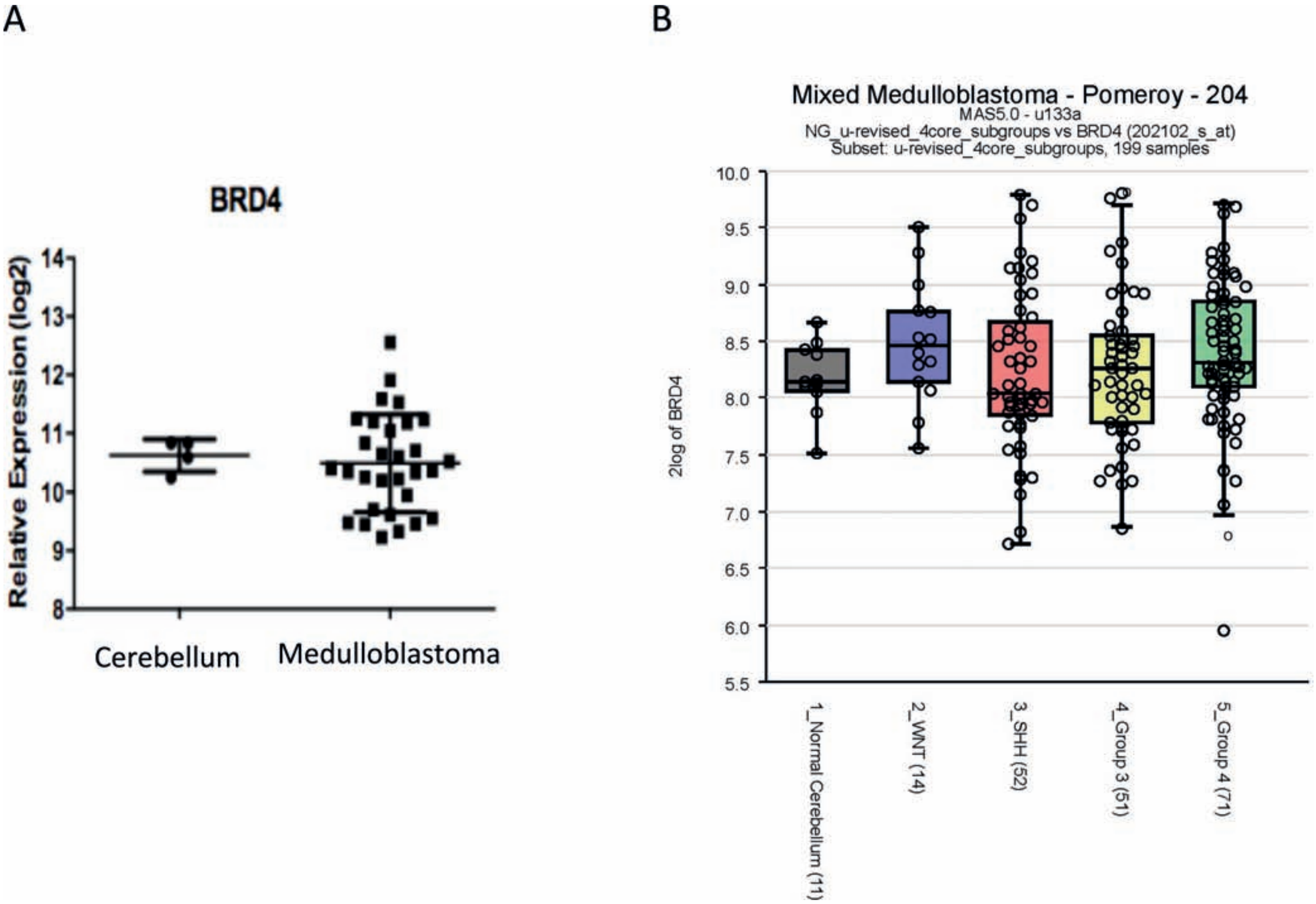

Supplementary Figure S2: Expression of BRD4 in medulloblastoma patient samples. **(A)** BRD4 mRNA expression in Colorado Patient cohort. **(B)** BRD4 mRNA expression by subgroup analysis in large data set from Cho et al (JCO 2012).

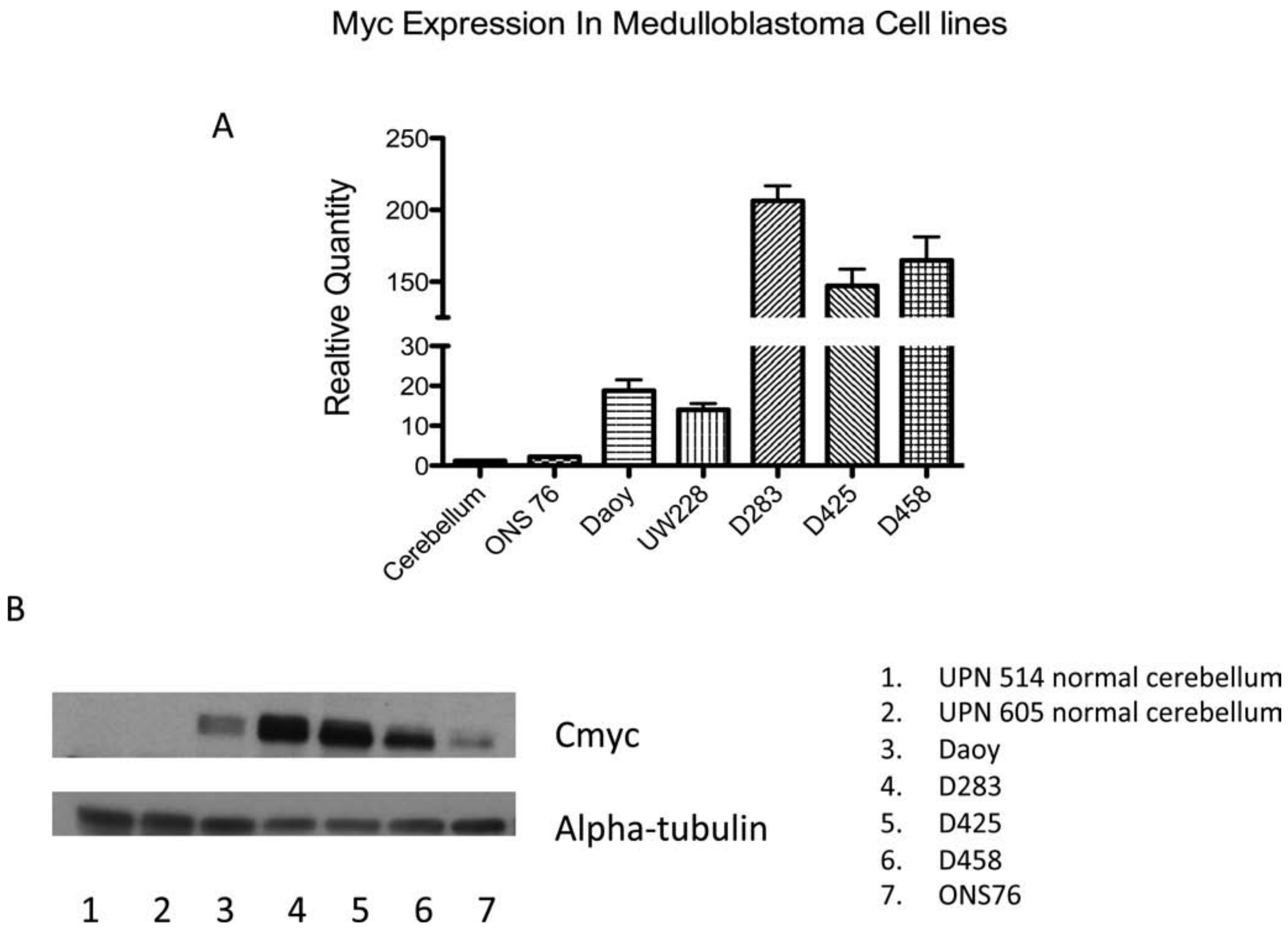

Supplementary Figure S3: (A) Expression of MYC mRNA in medulloblastoma cell lines used in functional studies. **(B)** Immunoblot analysis of MYC protein in normal cerebellum and medulloblastoma cell lines.

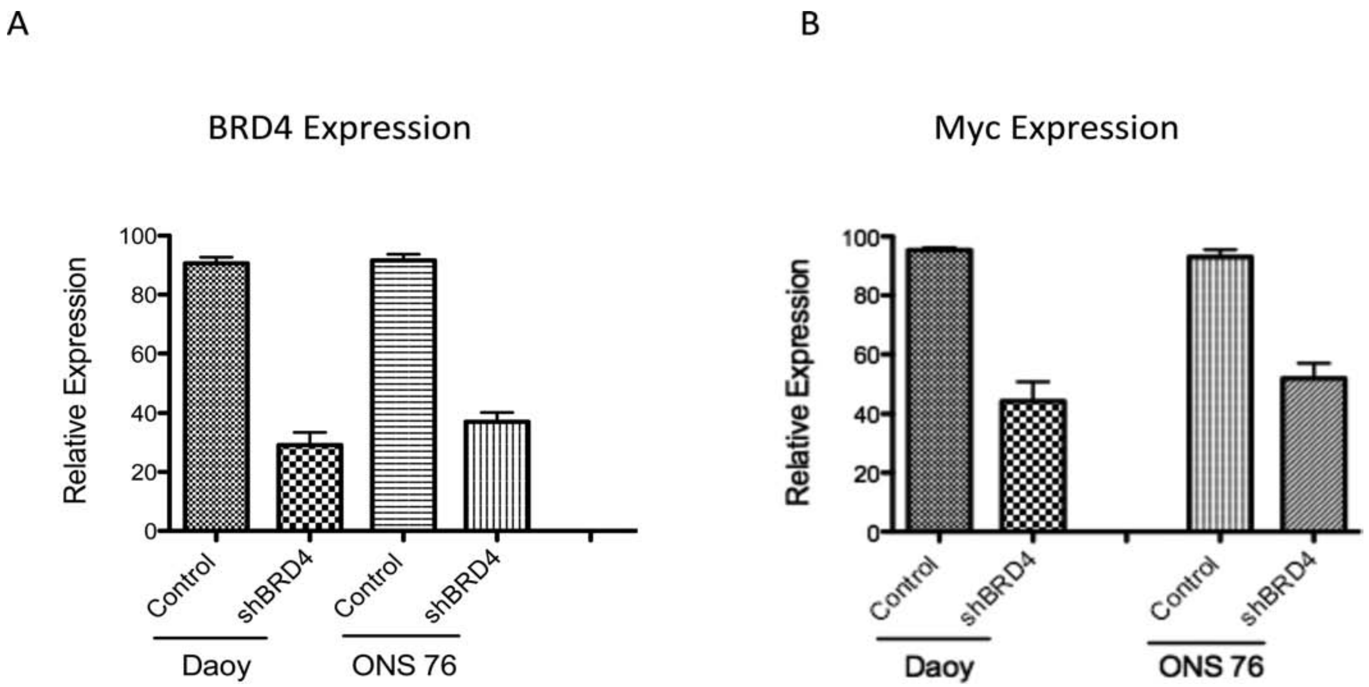

Supplementary Figure S4: Gene expression effects of BRD4 knock down with shRNA. (A) Expression of BRD4 mRNA in DAOY and ONS76 cells transfected with control vector or shBRD4. **(B)** Expression of c-MYC mRNA in DAOY and ONS76 cells transfected with control vector or shBRD4.

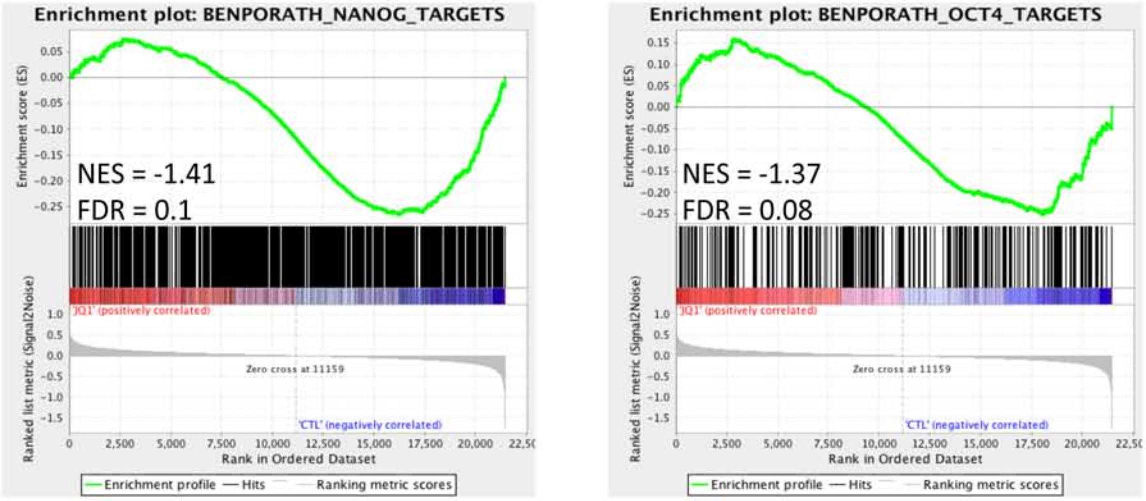

Supplementary Figure S5: JQ1 suppresses stem cell associated signaling in medulloblastoma cells. GSEA of Nanog and OCT4 dependent gene sets in transcriptional profiles of DAOY medulloblastoma cells treated (red) or untreated (blue) with JQ1.

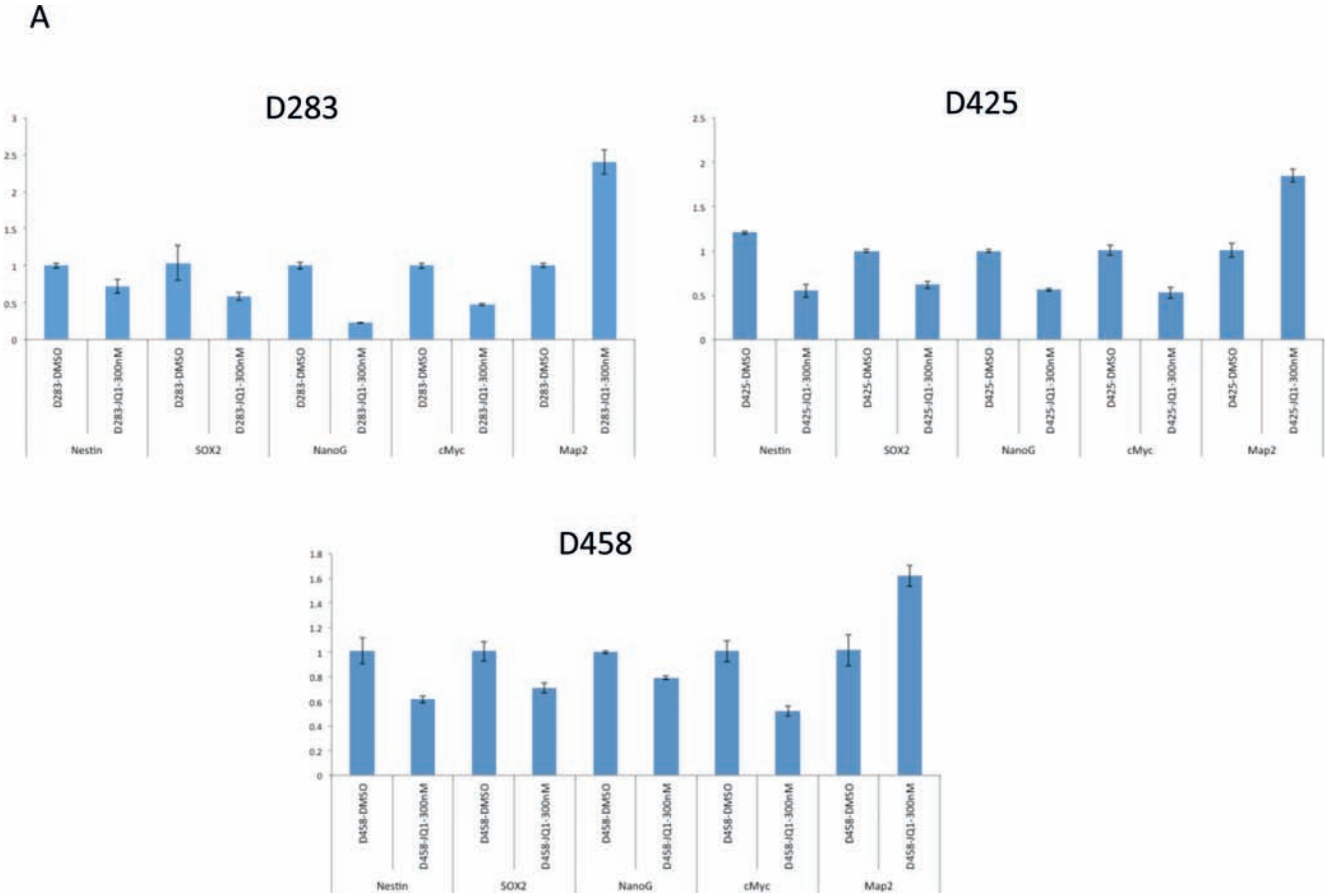

Supplementary Figure S6: Expression of stem cell associated markers (Nestin, Nanog, SOX2) and differentiation marker (MAP2) in D283, D425 and D458 medulloblastoma cells treated with 300nM JQ1 or control DMSO treated controls by qPCR analysis confirm results from DAOY cells.

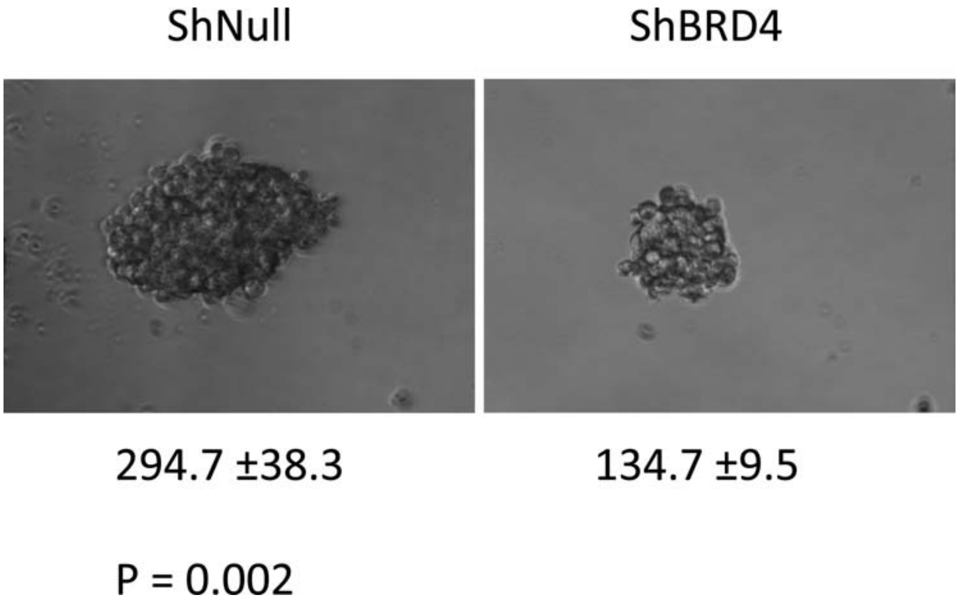

Supplementary Figure S7: Genetic inhibition of BRD4 with shRNA suppresses tumor sphere formation as seen by significantly decreased sphere size (n = 3); size measured in microns.

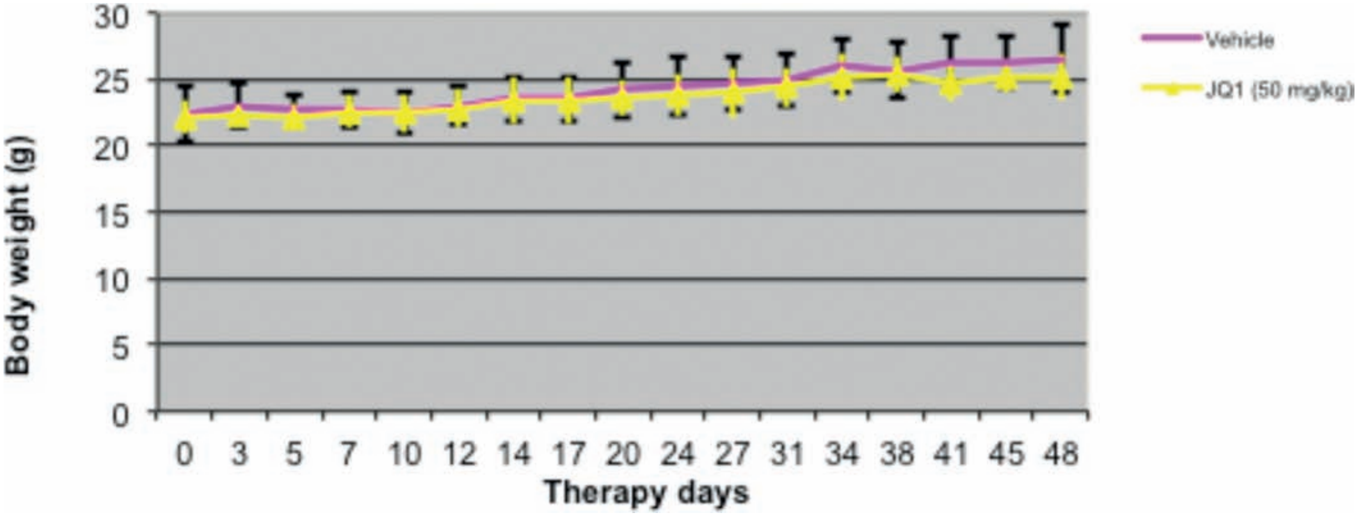

Supplementary Figure S8: Weights of control or JQ1 treated mice during *in vivo* xenograft study.

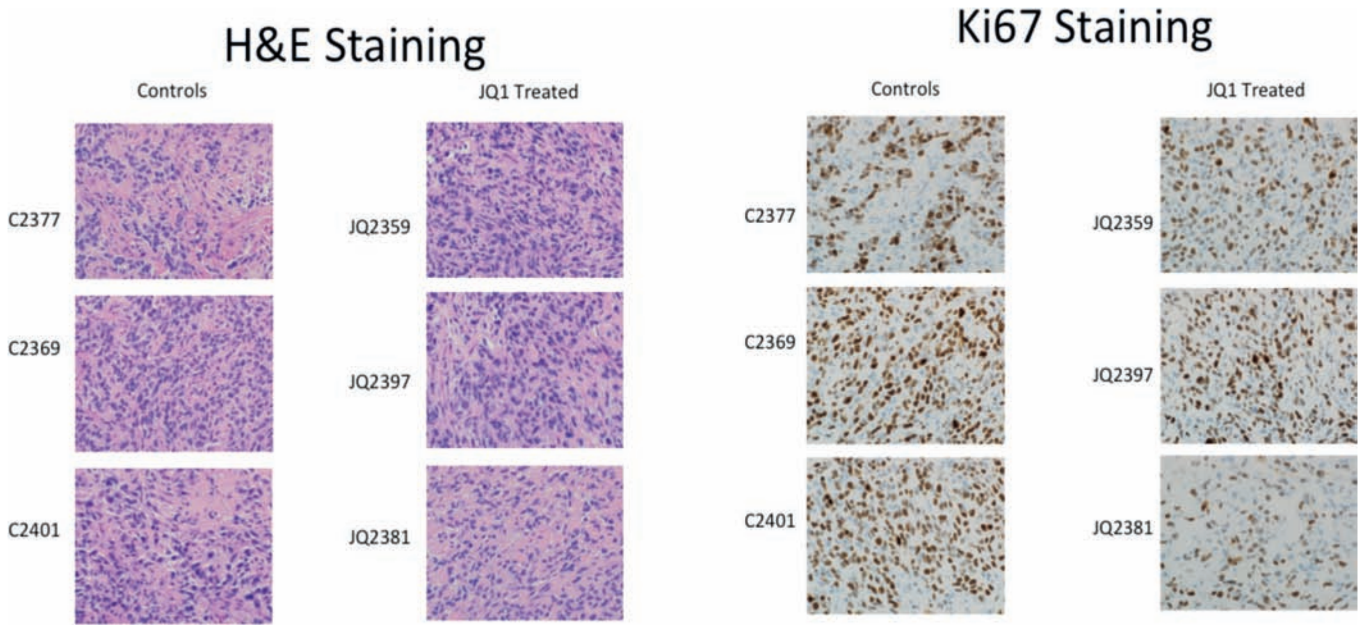

Supplementary Figure S9: H&E staining and Ki67 IHC of all three control and all three JQ1 (300nM) treated xenografts analyzed.

A

Gene
IFRD1
CRIP1
CENPF
CDC25B
BARD1
MYBL2

B

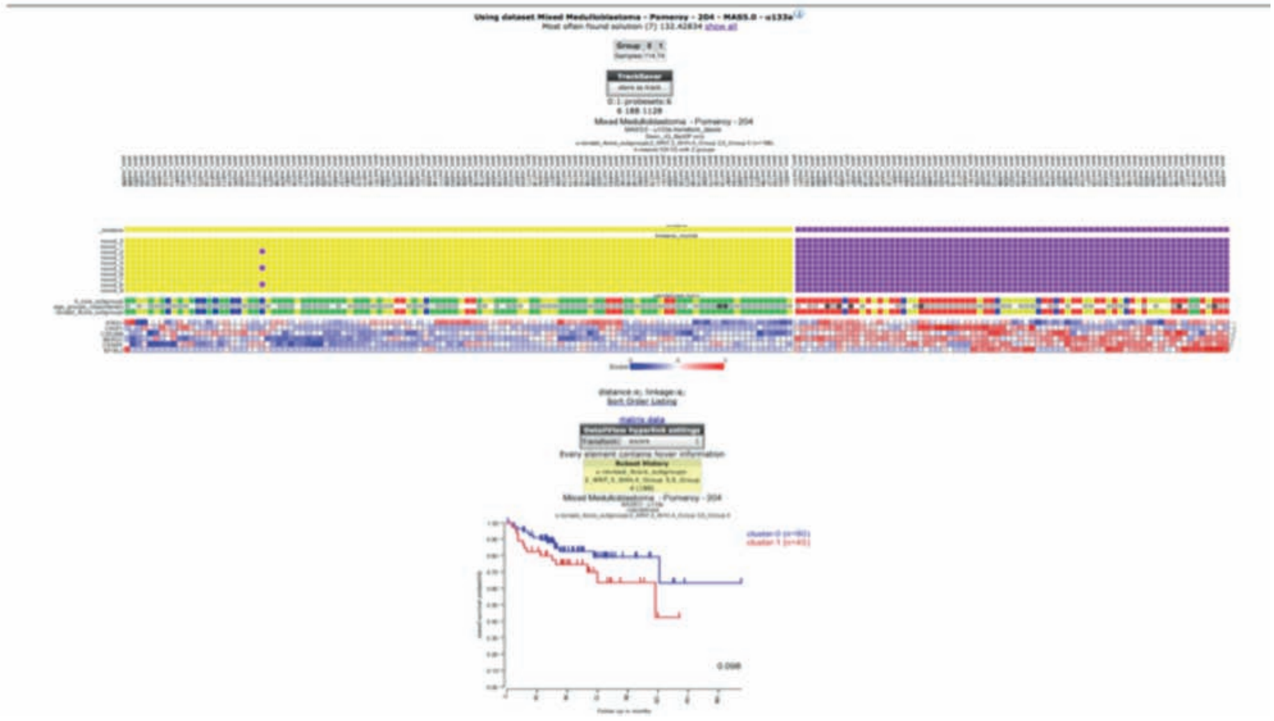

Supplementary Figure S10: (A) List of the six-gene signature **(B)** *k*-means clustering and kaplan-meir analysis of a 6 gene signature in a large cohort of medulloblastoma samples from Cho et al (2012).

Supplementary Table S1: List of genes altered in DAOY medulloblastoma cells by 300nM JQ1.

GENE	fold change (JQ1/DMSO)	p-value	FDR
TRAJ17	0.043593696	0.000262429	0.010007579
TNFSF4	0.086344016	1.68842E-05	0.003403309
PDCD1LG2	0.099232052	2.19632E-05	0.003661641
CCL2	0.101394408	1.89858E-07	0.001272452
IL7R	0.106598324	2.77327E-06	0.002229464
TRAJ23	0.108110858	0.000125047	0.007226592
HAS2	0.108673594	7.22737E-06	0.002575353
MIR142	0.116247103	0.000189497	0.008647792
ACTBL2	0.120339041	0.000279241	0.010346969
FLJ22447	0.12722846	3.12968E-07	0.001434447
EDN1	0.129824388	5.60185E-05	0.004873588
RHOJ	0.135076006	3.1132E-05	0.003974296
KCNJ15	0.143887184	5.21191E-08	0.001272452
TRBC2	0.150093492	3.3967E-05	0.004065425
PTPN22	0.161227901	0.00049062	0.013786988
ANXA3	0.166639617	2.61921E-05	0.003800098
PDGFRB	0.174151725	9.10296E-05	0.006098098
ODZ2	0.174359072	2.61772E-06	0.002229464
TNFSF10	0.181949921	0.000233794	0.009450269
FAM196B	0.187145639	1.1444E-05	0.002969775
SCARA3	0.201646928	4.1664E-05	0.004363083
NIPAL4	0.203432206	0.001091058	0.021714646
AMTN	0.211075836	3.20918E-05	0.003996833
GBP1	0.211670188	0.000405872	0.01249787
LOC152225	0.213206501	2.5879E-05	0.003800098
IL6	0.229587075	2.73522E-05	0.003810686
SERPINB2	0.235455011	1.68649E-05	0.003403309
MICALCL	0.243139579	3.82557E-05	0.004277602
IGFBP5	0.243278377	3.61786E-05	0.004153722
GPR87	0.244124106	0.000727862	0.017214723
LOC100216001	0.245880631	0.000168042	0.008265971
FAP	0.252699842	5.25893E-05	0.004771033
FAM19A3	0.253843871	0.000140609	0.007661461
VEPH1	0.259876845	9.00513E-05	0.006073309
FAR2	0.263241101	3.16779E-06	0.002230648

(*Continued*)

GENE	fold change (JQ1/DMSO)	p-value	FDR
LAMB3	0.266405665	2.07445E-05	0.003622055
TNS3	0.266554664	3.8532E-05	0.004277602
TLR3	0.268644461	0.000116486	0.007017562
CD274	0.269802072	0.000505369	0.014003513
KRT80	0.270417421	0.000233765	0.009450269
LRRC34	0.270940256	3.9157E-06	0.002358967
ACSL5	0.271944345	8.52209E-05	0.005903502
TGFB2	0.272348029	5.26481E-06	0.002520023
HHIP	0.273716315	1.20642E-05	0.003008587
RGS4	0.275943218	0.000271614	0.010205415
MPP4	0.27632283	9.06507E-05	0.006098098
CSF2	0.277394932	0.002627972	0.03644233
IL1A	0.281234079	0.000179588	0.008500179
LOC730755	0.282168074	0.00025315	0.009814276
LOC100509976	0.283443548	0.002840736	0.038067923
SERTAD4	0.284576151	0.00070143	0.01687997
HIST1H2BM	0.285512009	0.001121276	0.022013715
HTR1D	0.286698581	5.9492E-06	0.002575353
APOL6	0.28718056	0.000373567	0.012025592
IL8	0.288078387	8.45857E-05	0.005874652
ZNF532	0.288385393	1.65771E-07	0.001272452
NNMT	0.289045797	0.002019747	0.030854766
TEK	0.289904574	1.08406E-05	0.002935558
NAV3	0.290694699	4.63151E-05	0.004592806
LINC00460	0.292496243	0.000222857	0.00929053
E2F8	0.293163351	6.95137E-05	0.005369556
AK5	0.299497666	4.04269E-05	0.004347483
PRDM1	0.300472181	8.65069E-05	0.005950367
LOC100506178	0.301811566	0.001656147	0.027508561
SERPINB7	0.302323843	0.000133252	0.007473402
LOC100216479	0.303620267	0.000834928	0.01869158
KCNH1	0.305777325	7.63745E-05	0.005567586
IKBKE	0.307504622	1.05467E-05	0.002929961
NEXN	0.308150421	6.1705E-07	0.001586766
CTSS	0.311041718	0.00120765	0.022953056
GPX8	0.313660696	0.000666093	0.016505294
TNFAIP3	0.314109613	6.63811E-07	0.001586766
F2RL3	0.314153161	0.000681851	0.016685899

GENE	fold change (JQ1/DMSO)	p-value	FDR
ZNF474	0.316441342	2.58953E-05	0.003800098
FMNL1	0.317454118	1.16944E-05	0.002969775
TDO2	0.318906778	0.001123329	0.022045948
PDE1C	0.319546251	2.12773E-05	0.003629837
F3	0.320033906	6.37743E-06	0.002575353
STRA6	0.321366177	1.03681E-06	0.001684565
CLIC6	0.322094664	7.00356E-05	0.005369556
PLK2	0.323291317	4.04711E-06	0.002359839
PTPN7	0.32881925	0.000140749	0.007661461
LRRC17	0.334228501	0.00010333	0.00656324
LIF	0.339808379	2.73812E-05	0.003810686
AIM1	0.3426862	0.000207609	0.009061273
SH3RF2	0.344588549	6.4587E-05	0.005195423
LOC100507503	0.344721535	0.001951989	0.03025724
ABCC9	0.346746526	0.00028694	0.010499642
TNFRSF9	0.347232366	0.00048062	0.013663522
LOC541472	0.347929441	0.000102375	0.006559723
C15orf54	0.349337478	0.00016988	0.008283632
ANXA8L1	0.349596666	0.000120164	0.007142831
CTHRC1	0.351781942	6.64473E-06	0.002575353
LOC100507127	0.351996584	0.000122333	0.007180161
GBP3	0.35214626	0.001243489	0.023401948
TNFRSF8	0.353103575	0.00028426	0.010467835
GDF15	0.354857901	1.95731E-05	0.003569561
BHLHE41	0.355745316	0.000122399	0.007180161
CDK15	0.356093169	2.64472E-06	0.002229464
SMAGP	0.356450422	0.000676927	0.016618496
GRIK1-AS2	0.357607773	0.001191516	0.022783707
DCSTAMP	0.358000457	0.0005276	0.014374151
LOC100128130	0.358199857	0.002789715	0.037744041
FOXF2	0.358836861	4.49358E-05	0.004589186
TGFB1I1	0.359932097	0.000149866	0.007901048
SPOCD1	0.363451979	6.18604E-05	0.005079279
HIC1	0.364517504	3.21559E-05	0.003996833
ITGB6	0.365223964	0.003384701	0.042421111
CACNA2D3	0.36526447	0.00035331	0.011677142
ERAP2	0.365859933	7.69456E-07	0.001586766
TMEM156	0.366060328	0.000249703	0.009751265

(*Continued*)

GENE	fold change (JQ1/DMSO)	p-value	FDR
C1QTNF2	0.367134348	0.000651638	0.016326577
FAM83B	0.36778724	0.003015565	0.039377874
FBXL13	0.367931729	0.000796527	0.018150186
SH2D1B	0.369540229	0.000174217	0.008407734
WNT5A	0.369925502	2.34268E-05	0.003749477
LOC100507600	0.371120603	0.001743609	0.028303689
ANK3	0.373610438	0.000188493	0.008647792
TRGV5	0.37564453	0.002250777	0.032972653
PLAC8	0.375739146	6.71079E-05	0.005259719
IFI44	0.375894575	7.4027E-07	0.001586766
RAET1E	0.3781088	1.06862E-05	0.002935503
IL24	0.378335135	0.000413338	0.012619675
DDR2	0.378416439	0.000203811	0.008994373
MGLL	0.378490764	3.42702E-05	0.004083199
SORBS2	0.37853799	2.12829E-05	0.003629837
DSC2	0.379973313	1.43868E-05	0.00323775
LRRTM2	0.381009797	0.000518392	0.014224475
TUBBP5	0.382295467	0.003927377	0.046700859
ANGPTL2	0.384017333	0.000810627	0.018338982
ANXA8	0.386341885	0.001277865	0.023685722
CHRNA9	0.386664262	0.000132873	0.007473402
MLKL	0.386785782	0.000408691	0.01255747
SEMA3A	0.387540771	0.000164217	0.008228806
SLAMF7	0.38913878	0.001429441	0.02531957
PARP14	0.389440096	0.000117852	0.007060191
OAS3	0.390057846	1.61707E-06	0.002083338
POLE2	0.390781299	1.57237E-05	0.003303415
OTOGL	0.391127261	0.000169946	0.008283632
TNS1	0.391431926	0.000986464	0.020586842
EMP3	0.393468487	1.9856E-06	0.002129238
IL17RB	0.394403247	0.000927598	0.019911381
CLMP	0.396039619	0.000234243	0.009450269
JAG1	0.396084459	6.41918E-08	0.001272452
LYPD1	0.396219924	0.000167256	0.008265971
RUNX3	0.397146538	0.000359049	0.011745656
BDKRB1	0.399620731	0.000519303	0.014242184
TFPI	0.401305639	0.00087342	0.019255822
VCAN	0.40208897	0.000500177	0.013974982

GENE	fold change (JQ1/DMSO)	p-value	FDR
ZBTB2	0.403818805	6.57045E-06	0.002575353
LAT	0.403911184	8.93034E-05	0.006045043
LPXN	0.405192675	2.32778E-05	0.003745677
SLC7A11	0.4067535	1.82749E-05	0.003510063
SIRPA	0.407201091	0.000102402	0.006559723
LRRN3	0.407903559	0.002596442	0.036168727
GIPC3	0.407954455	1.03382E-05	0.002925673
PHF19	0.408756431	4.6919E-06	0.002378207
MT1A	0.413704723	1.03353E-05	0.002925673
GRPR	0.414268108	0.003146867	0.040395115
TMEM204	0.41549989	0.001856915	0.029367601
ZNF618	0.415536372	5.68296E-05	0.004873588
BRIP1	0.416259951	1.90394E-06	0.002083338
TGM2	0.417804518	5.20748E-05	0.004750001
SYNC	0.417865339	6.36272E-05	0.005155428
ZPLD1	0.418833864	0.002371769	0.034032566
TMEM154	0.420036518	1.52907E-05	0.003268202
RAB39A	0.421133632	0.001153694	0.022379744
PTX3	0.421170609	3.48028E-05	0.004083199
MYO5C	0.42418044	0.000175631	0.008430445
TM4SF1	0.425096809	3.30847E-05	0.004031596
PHF15	0.425220582	3.32208E-05	0.004039001
NLRP3	0.426556869	0.000567613	0.015014162
PPAPDC1A	0.427362828	0.000213471	0.009163871
IFI44L	0.427460594	0.000148803	0.007883765
SNTB1	0.427791582	1.60071E-05	0.003326561
CDA	0.42811986	0.000547093	0.014681424
SSX4	0.42829003	0.001400262	0.025005798
HAPLN1	0.429690564	0.000458816	0.013339328
DHRS4L2	0.429861359	0.002328854	0.033701221
XDH	0.430425861	0.001954921	0.030262224
LACE1	0.430959241	6.84418E-05	0.005318325
KLF11	0.431485305	3.21757E-05	0.003996833
ICAM1	0.433248502	0.00017159	0.008341016
DRP2	0.434883236	0.000502054	0.013986545
RIN2	0.436950977	0.000320903	0.011115243
LINC00470	0.437590503	0.003957873	0.046869924
BRCA1	0.438356537	1.17424E-05	0.002969775

(*Continued*)

GENE	fold change (JQ1/DMSO)	p-value	FDR
SCG5	0.438660488	0.001879982	0.029568494
TMEM194B	0.438827751	8.04725E-06	0.002661222
ATP8B1	0.439078257	3.40777E-06	0.002255733
PIK3CG	0.440194594	6.88527E-06	0.002575353
ST6GAL2	0.441057911	7.35207E-06	0.002575353
BIN1	0.441370874	3.86305E-05	0.004277602
MCTP1	0.44196785	0.000214657	0.009178042
MYCT1	0.443150916	0.000538769	0.014560069
SIGLEC15	0.443356767	3.19576E-05	0.003996833
SLCO2A1	0.443910273	1.7949E-06	0.002083338
CEP41	0.444329962	0.00032662	0.011182925
S1PR1	0.446008569	0.000806127	0.018272139
PRTFDC1	0.446376609	4.41548E-06	0.002367448
SLC16A9	0.44664484	0.000413352	0.012619675
NRG1	0.447008241	7.03931E-05	0.005369556
XRCC4	0.447458772	0.000341387	0.011437601
TMEM45A	0.447566305	0.000516425	0.014213017
CDH11	0.447801107	3.33978E-06	0.002255733
PCSK1	0.448663792	0.000509877	0.014091791
VAMP8	0.449014311	1.3605E-05	0.003213477
EPHX4	0.449063074	8.74326E-08	0.001272452
WDR76	0.450459716	4.56532E-06	0.002376493
SYNPO	0.450759562	0.00128561	0.023769156
CSF1	0.450930396	0.000156531	0.008039006
GINS2	0.452287909	8.28519E-06	0.00268001
KCNH1-IT1	0.452353749	0.0038278	0.04594474
ROS1	0.453339361	3.75798E-05	0.004258072
CLCN4	0.453414783	0.000221298	0.009276327
PRR16	0.453499647	8.59947E-05	0.005941724
MYEOV	0.453985041	1.41833E-05	0.00323775
COL6A3	0.454285134	6.51353E-05	0.005201687
AMIGO2	0.454360713	2.04392E-05	0.003622055
MGC11082	0.454961593	0.000245384	0.009685506
RAMP2	0.455131917	0.00134065	0.024408024
LCP1	0.455707494	1.79756E-05	0.003504171
MPZL3	0.455728552	0.001562322	0.026702907
LOC643650	0.45604771	0.002535792	0.03562934
HIST1H2AE	0.456219494	0.000460317	0.013362174

GENE	fold change (JQ1/DMSO)	p-value	FDR
PLEKHA2	0.456223711	0.000780122	0.017972842
BZRAP1-AS1	0.456324916	0.000522218	0.014285593
LUM	0.456896723	0.000804589	0.018265964
THBS1	0.456927338	3.87859E-05	0.004277602
PCDHB7	0.457959948	0.00025408	0.009843213
CHRDL1	0.458206554	1.26684E-05	0.003073491
GBP4	0.458422576	0.002047052	0.031119021
RAD51AP1	0.458462827	1.16308E-06	0.001834143
IFIT3	0.459502092	0.00016344	0.008205961
KYNU	0.460119339	0.002917143	0.038679588
RASSF5	0.460509663	0.000463291	0.013427175
NFIC	0.460893928	2.5352E-05	0.003800098
MND1	0.461305159	0.000230641	0.009428262
SCN2A	0.462226966	0.001450836	0.025596491
SGOL1	0.464314208	0.000340017	0.011422739
HDAC9	0.46437858	0.000212809	0.009159657
CDCA7	0.466459372	0.000785069	0.018019283
TP73	0.467000716	6.232E-05	0.005102377
CUBN	0.467139928	0.004103787	0.04782281
ADAMTS1	0.468435829	0.000676439	0.016614123
C1QTNF9B-AS1	0.468484536	0.000177212	0.00848355
TMEM194A	0.468537578	4.02881E-05	0.004347483
LY6K	0.468539743	6.03523E-05	0.004995458
CPA4	0.468616611	1.61322E-05	0.003339615
GLT8D2	0.469593171	0.000195462	0.00875434
GPR183	0.470089274	7.00342E-05	0.005369556
RGCC	0.470697904	0.003471352	0.043127021
SCARF1	0.4731108	0.002350176	0.033877483
FLT3LG	0.474474788	0.000451629	0.01322017
FRMD4A	0.474724803	1.56443E-05	0.003303415
IKZF3	0.475360302	0.003445341	0.042900336
DCLK1	0.475696506	0.00135059	0.024497491
NDC80	0.475755861	0.000393557	0.012318357
PAPPA	0.476035147	0.000929698	0.019931075
MBNL3	0.476148447	0.000182414	0.008512177
MICAL2	0.47616825	5.38611E-07	0.001586766
TENC1	0.476988592	0.000266233	0.010102346
MTBP	0.477132986	4.85806E-05	0.004656499

(*Continued*)

GENE	fold change (JQ1/DMSO)	p-value	FDR
CDCP1	0.478521837	5.50337E-06	0.002522548
BIRC3	0.478555006	0.004054348	0.047546364
TFAP4	0.479163526	0.001332468	0.02432514
ALG14	0.479212241	8.31916E-05	0.005830698
TSPAN2	0.479266498	0.000230884	0.009428262
ADAMTS5	0.47977282	0.000825775	0.018556403
DNM3OS	0.479932472	0.000395702	0.012363843
SYK	0.480145424	0.000114865	0.006981504
LOC149773	0.482158705	0.002685803	0.036893481
CTTNBP2	0.484437796	0.001110571	0.021915894
NTM	0.485012331	0.004213391	0.048634959
MCM5	0.485181574	2.57509E-05	0.003800098
KIF18B	0.485410313	0.000124101	0.007216119
FAM69B	0.485607744	3.74429E-06	0.002307559
SSX4B	0.48651516	0.000182902	0.008527527
SPC25	0.487449049	5.61964E-05	0.004873588
DMRT2	0.48786932	0.002720492	0.037100455
BATF3	0.488036177	0.000115437	0.006996142
SLC43A3	0.48959249	8.65637E-05	0.005950367
GLIPR1	0.489742963	2.66803E-05	0.003800098
ADCY7	0.489861789	0.001217083	0.023075085
INHBA	0.489937627	1.87363E-05	0.003537268
RAD51	0.490190127	0.000288864	0.010543241
GYPC	0.490782825	0.002675887	0.036825727
ZNF689	0.490931394	0.001671225	0.027639134
MIR4755	0.491616985	0.001632325	0.027358665
E2F2	0.491695367	8.19876E-05	0.005787141
DTX3L	0.491868078	0.000272227	0.010206489
SNORD116-21	0.492554977	0.00068467	0.016726941
TMEM178A	0.492775807	0.002665604	0.036759617
ALDH1B1	0.493223462	4.08884E-05	0.004358476
DCLK2	0.493461693	0.001230348	0.023240599
HOXD13	0.493467394	6.37904E-06	0.002575353
ZMYND8	0.493854057	1.73314E-05	0.003454489
SRGN	0.494656863	1.7881E-06	0.002083338
MIR221	0.494842048	0.001503819	0.02617016
LOC100128816	0.494843191	0.00217188	0.032212916
CD101	0.495938569	0.002798278	0.037801782

GENE	fold change (JQ1/DMSO)	p-value	FDR
EBI3	0.496257221	0.001766172	0.028540339
S100A3	0.497046707	0.000160505	0.008134023
FAM72C	0.498254014	0.001643985	0.027473501
GPR68	0.4987827	0.00038372	0.012171722
BORA	0.500673962	0.000391844	0.012293446
IFIH1	0.501101004	0.002141362	0.031954747
LMNB1	0.501163528	0.000124627	0.007216119
TRIP6	0.501552745	1.50716E-05	0.003258443
HMCN1	0.501789203	0.004222555	0.048696248
LOC100190938	0.502036211	0.000346108	0.011519102
IFITM2	0.502351817	5.00406E-05	0.004692392
TMEM244	0.50249344	0.003763406	0.045344391
CGB2	0.50421933	0.003357266	0.042195624
GPR115	0.504687876	0.003362485	0.042241415
FAM176A	0.504766009	0.000312687	0.010993665
ALKBH8	0.504827824	3.3446E-05	0.004057181
APOBEC3C	0.504867483	0.001266147	0.023555518
MDFIC	0.504925811	0.000633219	0.016024825
LOXL3	0.505305107	0.000306335	0.010907855
SEMA6B	0.505578377	0.001016615	0.020884232
CFI	0.505656648	0.001074287	0.02144674
AOX1	0.506056369	0.002321692	0.033652922
F2RL2	0.50671742	1.04485E-05	0.002925673
BNC2	0.50674552	4.92231E-05	0.004662889
HSD17B2	0.506790013	0.001117134	0.021999746
SLC10A4	0.507507131	0.001243339	0.023401948
HJURP	0.507644343	6.34875E-05	0.005155428
NRP1	0.508355623	5.69885E-06	0.002522548
CACNG4	0.508675201	0.00102344	0.020944192
TXNRD2	0.508967932	0.000157283	0.008056377
UBXN8	0.509030262	5.46039E-05	0.004815292
C19orf40	0.509327905	0.001184534	0.022714956
MYLK2	0.50960335	0.000800659	0.018197937
DENND2A	0.509729351	0.001199695	0.022864449
PDGFC	0.51099934	0.000740839	0.017398846
ELOVL2	0.511533277	2.5619E-05	0.003800098
GINS1	0.512547162	1.11204E-05	0.002963198
CHD3	0.512925073	7.64262E-05	0.005567586

(Continued)

GENE	fold change (JQ1/DMSO)	p-value	FDR
SFXN2	0.512978405	0.000228016	0.009368225
UBE2C	0.512983146	2.77675E-06	0.002229464
CFLAR	0.514242232	5.80093E-05	0.00493696
NRK	0.515121026	0.002963727	0.038966687
NKX2-2	0.515883306	0.000473061	0.013541971
PCDHB5	0.516708791	2.33051E-06	0.002229464
HS6ST2	0.51700734	0.000342706	0.011439572
ETV4	0.517327576	1.67971E-05	0.003403309
NEK3	0.517429184	2.52446E-05	0.003800098
ETV6	0.517480594	0.000294569	0.010671558
LOC100507516	0.518255945	0.00225279	0.032991732
CNRIP1	0.518748321	3.36349E-05	0.004061922
KIAA1644	0.518922142	0.002926548	0.038695826
ESPL1	0.518982093	1.44759E-05	0.00323775
GMFG	0.519581991	0.000153203	0.00799833
CENPQ	0.520598599	0.004094873	0.047760795
MNS1	0.520782665	0.000223786	0.00931247
GTF3C6	0.521442471	4.79039E-06	0.002378207
CENPK	0.52158104	4.76066E-06	0.002378207
C3AR1	0.522565336	0.000508232	0.014053572
SNAI2	0.522948217	4.26925E-06	0.002359839
PSRC1	0.522969966	0.003873585	0.046301899
C1orf31	0.523216521	0.000189572	0.008647792
DKK2	0.523303568	0.003258525	0.041423595
PBK	0.523474077	1.19256E-05	0.003001948
MARCH4	0.52366884	0.000252618	0.009803337
PTPRB	0.524111862	0.001788671	0.028739981
FANCD2	0.52445831	0.000214053	0.009174164
PLK4	0.524618286	0.004028732	0.047359904
NET1	0.524885021	9.60418E-06	0.002892962
BCAR3	0.526507748	0.001052169	0.021232736
DEPDC1	0.526902039	3.24267E-05	0.003996833
ARHGAP11B	0.52738435	2.64601E-05	0.003800098
CC2D2A	0.529029471	0.002739825	0.037273526
SPP1	0.529083256	0.003265272	0.041443999
C15orf48	0.529151717	0.001524176	0.026319402
GRAMD1B	0.529161498	1.08213E-05	0.002935558
HOXB5	0.529198178	0.002320238	0.033650041

GENE	fold change (JQ1/DMSO)	p-value	FDR
MYLK	0.529658116	0.000952392	0.020191539
PPAP2C	0.530566932	1.04767E-05	0.002925673
FRMD4B	0.53111886	0.002059812	0.031242133
MIR143	0.531316468	0.000890128	0.019448835
PALMD	0.532001911	2.89505E-05	0.003813855
SHC4	0.53205231	0.000903629	0.019601764
CALD1	0.532631626	7.46778E-05	0.005529648
SH2D2A	0.533145051	0.000329855	0.011250532
SYDE1	0.533201718	7.89014E-05	0.00565569
LOC100287314	0.533926601	0.000977432	0.020469991
SNORD91B	0.534402996	0.003945356	0.046810833
ZNF367	0.53471671	0.000133107	0.007473402
GEM	0.53481432	2.61737E-05	0.003800098
TM4SF19	0.53529027	0.00015732	0.008056377
BANK1	0.536384703	0.000355325	0.011695146
KRT34	0.536702061	0.000525567	0.01433333
CCDC34	0.537270302	0.000180979	0.008500497
LGALSL	0.538177258	0.000175211	0.008417821
PBX2	0.538481991	2.43922E-05	0.003758137
FMO3	0.538516828	0.002728716	0.03717113
PCED1B	0.538549179	0.000659936	0.016434644
CDK1	0.538977393	0.000779895	0.017972842
MCM2	0.539101938	1.14418E-05	0.002969775
MMACHC	0.539889726	0.001570929	0.02672224
KDM4D	0.54024161	1.02789E-05	0.002925673
RIBC2	0.540285299	0.002693595	0.0369556
CXCL10	0.540304025	0.003535665	0.043700265
KLHDC8A	0.540396412	0.001655896	0.027508561
SRPX2	0.540852336	0.000958157	0.020265682
PRR11	0.54129989	6.38547E-05	0.005163948
SNORA45	0.542201121	0.003606737	0.044262398
HMGA2	0.542257497	8.2974E-06	0.00268001
SNORD123	0.542331422	0.003160502	0.040509854
FHL2	0.543250687	7.23169E-05	0.005438644
PCDHB15	0.543374964	0.001905844	0.029811583
MORN4	0.543734145	7.05851E-06	0.002575353
CKAP2L	0.543751733	7.23232E-05	0.005438644
HOXD10	0.543869842	0.001478313	0.025893494

(*Continued*)

GENE	fold change (JQ1/DMSO)	p-value	FDR
MCPH1	0.5445438	2.76266E-05	0.003810686
C4orf19	0.544753953	0.001059061	0.021307195
EIF2D	0.544996927	0.001050423	0.021213006
EFEMP1	0.545063669	4.70566E-07	0.001586766
AURKA	0.545271504	0.000518059	0.014224475
PRSS23	0.5453156	0.000356312	0.011698947
SUV39H1	0.545561345	0.000276423	0.010285199
CD3D	0.545789546	0.00271624	0.037097844
MOK	0.545977474	2.37546E-05	0.003758137
TRBV20-1	0.546297982	0.001143427	0.022244966
FBXO5	0.546339637	8.8106E-05	0.006033179
MCM10	0.546343424	0.000322972	0.011136864
ANKRD36B	0.54684985	0.002229162	0.032736505
SNORD13P3	0.546921873	0.003351733	0.042172651
METTL4	0.547414922	0.002593041	0.036131239
PBX2	0.548052751	2.85126E-05	0.003810686
PBX2	0.548052751	2.85126E-05	0.003810686
ANO1	0.548161661	0.000474493	0.01355556
AHSA2	0.548898005	0.003409241	0.042599225
TMPO	0.549130139	5.0973E-05	0.004716623
WEE1	0.549405529	0.000141506	0.007671514
IL31RA	0.54947408	0.000275011	0.010239767
HOXA4	0.550429615	0.000143895	0.007746203
ARHGAP11A	0.550844366	4.53495E-06	0.002376493
CD82	0.550913097	5.6477E-05	0.004873588
DDX60	0.551067137	0.002305563	0.033573431
TRBJ2-3	0.551133349	0.00134911	0.024491353
MB21D1	0.551289998	0.000258908	0.009936915
LOC100134259	0.552309941	0.000688098	0.016769887
RPL29	0.552589479	5.43389E-06	0.002522548
RFC3	0.552677582	4.64674E-06	0.002378207
FOXM1	0.552712061	9.08902E-05	0.006098098
MBOAT1	0.552954751	0.000841544	0.018776995
NHLRC3	0.554182605	0.001475824	0.025870527
LOC286297	0.554351648	0.000163883	0.008219752
PLEKHG4	0.55459634	1.2314E-05	0.003021453
PBX2	0.555499172	8.00277E-06	0.002661222
PBX2	0.555499172	8.00277E-06	0.002661222

GENE	fold change (JQ1/DMSO)	p-value	FDR
PBX2	0.555499172	8.00277E-06	0.002661222
CALB2	0.555598009	0.002236356	0.032824172
FRMD6	0.555689159	4.62143E-05	0.004592806
MKX	0.556020508	0.001722026	0.028043222
TOMM40L	0.556071897	1.74225E-05	0.003459786
ANXA8L2	0.556947534	4.38866E-06	0.002367448
SAMD9L	0.557062073	0.000466638	0.013480458
C5orf34	0.557094251	0.000970566	0.020431387
RAD54L	0.557116133	0.003922839	0.046672189
VAX1	0.557129006	0.003265772	0.041443999
PLBD1	0.557217832	4.87333E-05	0.004656499
WDR66	0.557565551	0.00278264	0.037704526
FAM200B	0.55764414	0.000901678	0.019588845
ZNF93	0.558349358	0.003130942	0.040251806
BAIAP2L1	0.558634534	0.000297155	0.010714566
SFXN4	0.558777822	0.000622563	0.015849934
GPATCH4	0.558839796	7.97366E-05	0.005695459
FAM225A	0.558882407	2.44446E-05	0.003758137
H2AFY2	0.558910816	0.000108469	0.006723448
DOCK2	0.559565918	0.000206961	0.009058472
CMPK2	0.559690048	0.000587127	0.015318729
KLF7	0.559775403	3.67925E-05	0.004197241
BDH2	0.559846542	0.000604598	0.015569996
CDCA3	0.560528642	0.000168828	0.008281033
LOC375295	0.560782539	0.000675791	0.016613428
METAP1D	0.56100544	0.000317233	0.011073621
NEIL3	0.562014789	0.000131624	0.007444562
TIPIN	0.562821755	1.40027E-05	0.00323775
TMEM79	0.563382504	0.00102872	0.021005521
TBL1XR1	0.563437178	0.000234691	0.009454115
LIMS1	0.56432702	0.000565581	0.015007996
TMEM158	0.564971501	0.00020449	0.008994373
TTLL11-IT1	0.56500283	0.00169422	0.027847638
TAGLN	0.565589274	0.001767277	0.028542348
GPR39	0.565876841	0.000171955	0.008351188
MLPH	0.566016756	0.001120736	0.022013715
PRIM1	0.566363423	1.03982E-05	0.002925673
CDC45	0.566650074	8.88445E-05	0.006036779

(*Continued*)

GENE	fold change (JQ1/DMSO)	p-value	FDR
EMP2	0.568320502	0.001781507	0.028684403
EPB41L3	0.569181233	2.26795E-06	0.002210921
SNORD114-15	0.569448259	0.000702673	0.016889235
CNN2	0.56980361	6.77924E-06	0.002575353
ME3	0.570065659	0.00148632	0.02594986
SEMA3E	0.570571662	0.002609223	0.036274187
ANGPT1	0.570692958	0.003124897	0.040212711
MSC	0.571008186	3.21999E-05	0.003996833
FAM111A	0.571107143	0.001301831	0.023922571
ITGA4	0.571196878	0.00014831	0.007865418
SHROOM2	0.571336788	0.001674941	0.02767498
PLEKHO1	0.57146749	0.000545521	0.014653908
KCNQ3	0.571534833	0.000657658	0.016416038
CD70	0.571558603	0.000339287	0.011405361
ZBTB38	0.571682751	0.001284313	0.023753367
TNC	0.571685393	1.67994E-05	0.003403309
ZFP36L2	0.572088401	0.000668179	0.016524492
BCAT1	0.572381917	1.42875E-05	0.00323775
CCNE2	0.572515504	0.000184915	0.008547613
TMEM144	0.572606783	0.000635355	0.016024825
KIRREL3	0.572904536	0.000856119	0.019023014
USP1	0.572928363	0.000673893	0.016599178
IGFBP7	0.573276614	0.000129574	0.007383762
SP100	0.574465968	0.000753442	0.017594643
C4orf26	0.575655129	0.000293067	0.010653134
TNIP1	0.575734937	0.000515805	0.014204374
PCDHB6	0.576208694	0.002962866	0.038964922
COL8A1	0.576496332	0.004337956	0.049465799
LRRCC1	0.576940055	0.001653454	0.027508561
SLC20A1	0.576958717	0.000130912	0.007419777
MMS22L	0.577017375	0.000118789	0.007100457
FAM54A	0.577202719	0.000988212	0.020591218
IFITM4P	0.577809835	0.000286802	0.010499642
IFITM4P	0.577809835	0.000286802	0.010499642
CDC25A	0.578154374	0.00070012	0.016871161
TMEM145	0.578400218	0.000231378	0.009435328
ACOT2	0.578430955	0.000530003	0.014417641
LPAR1	0.579062108	0.000159296	0.008103391

GENE	fold change (JQ1/DMSO)	p-value	FDR
LOC81691	0.57919993	0.001339492	0.024395225
SLC46A3	0.580351957	1.57291E-05	0.003303415
CD3EAP	0.580828171	0.001377872	0.024782745
ADAMTSL1	0.58086709	0.000334515	0.011337352
MIR548H3	0.581093948	0.001198676	0.02286354
RNF144A-AS1	0.581222853	0.001740976	0.028269506
DLG4	0.581449849	4.33865E-05	0.004472203
ATP10A	0.581799247	0.00069023	0.016798939
CENPL	0.582062778	0.000198495	0.008846805
PRRG4	0.58267366	0.000887376	0.019419771
S100A2	0.582859474	0.003126798	0.040223015
VPS52	0.582878328	1.07309E-05	0.002935503
COL27A1	0.583635685	0.002789068	0.037744041
TNF	0.583655912	3.21043E-07	0.001434447
SERPINB5	0.583719297	0.003935928	0.046747896
AAED1	0.584128089	0.000227786	0.009365952
TRIP13	0.584799237	2.02889E-05	0.003617099
LTBP2	0.584837071	0.001538352	0.026487418
TRAF5	0.585060071	3.36366E-05	0.004061922
TCF19	0.585786425	0.000190743	0.008647792
C9orf100	0.585913664	0.004061469	0.047601699
PRLR	0.586211564	0.003756551	0.045302518
SHCBP1	0.586307737	0.000107786	0.006704364
MELK	0.586673609	0.000151102	0.007935001
PLAU	0.586792905	0.000239606	0.009558746
PPP1R13L	0.586975964	0.002309986	0.033607109
MR1	0.587320541	0.001073617	0.02144674
TRPA1	0.587465757	0.001236519	0.023328093
TRERF1	0.587939658	0.003125247	0.040212711
PPEF1	0.588291597	0.003790269	0.045606565
SH3RF3	0.58832286	0.000383228	0.012171722
ERAP1	0.588329657	0.000764698	0.017744536
PIGC	0.588843708	0.004022487	0.047317614
IL1RAPL1	0.589011075	0.004212912	0.048634959
PARP2	0.589352761	0.000125113	0.007226592
RAB3IL1	0.589585657	0.00046406	0.013434938
KIF1A	0.590087172	3.78983E-05	0.004277602
KCNQ5-IT1	0.590331269	0.001092329	0.021717701

(*Continued*)

GENE	fold change (JQ1/DMSO)	p-value	FDR
EGF	0.590504517	0.001299428	0.023892809
TRIP10	0.590638239	0.000667528	0.016518836
SASS6	0.590718759	4.89737E-05	0.004656499
MTRF1	0.590888025	0.000215802	0.009204977
PKMYT1	0.591219872	0.001458191	0.025676134
PRC1	0.591349657	6.50527E-05	0.005201687
ZNF692	0.591430274	0.000233885	0.009450269
SMOC1	0.591545071	0.001696625	0.02787008
ARHGAP31	0.59163255	0.000294259	0.010667535
TTPA	0.592353378	0.000239016	0.009556541
FERMT3	0.592390332	0.001791654	0.028746621
SKP2	0.592536802	2.16296E-05	0.003658405
CDC42EP1	0.592886013	0.001439103	0.025440285
LOX	0.593260102	6.59186E-05	0.005212917
NIPAL2	0.593489057	0.002923891	0.038695826
PRR5L	0.593560366	0.000371208	0.011994194
ZFP42	0.593965071	0.000389612	0.012252098
ARHGDIB	0.594028203	0.000485672	0.013763359
GPX7	0.594202536	3.51151E-05	0.004086104
TCF4	0.59453487	0.001398521	0.024986505
SNUPN	0.594868765	0.000470103	0.01351502
ITPR3	0.595154717	7.79084E-05	0.005607
POSTN	0.595180844	0.000490447	0.013786988
SHC3	0.595264735	5.01791E-05	0.004692392
CTPS2	0.595625187	0.000661148	0.016454224
MDFI	0.595877082	0.004265233	0.048948844
MFAP2	0.595889473	2.82258E-05	0.003810686
CAV1	0.596232394	6.29895E-06	0.002575353
LOC100506394	0.596268213	0.001918677	0.029941069
TSKU	0.596346745	4.67934E-05	0.004620482
CCDC80	0.596703716	0.000832802	0.018659567
TFR2	0.597066419	0.00041402	0.012619675
C21orf88	0.59753978	5.87582E-05	0.004947862
MYPN	0.597608815	0.000374688	0.012051096
ADTRP	0.597653001	0.003737854	0.045188843
FANCI	0.597867075	0.000227286	0.009352854
LOC283624	0.598016281	0.000245537	0.009685506
MMP1	0.598468271	0.001073493	0.02144674

GENE	fold change (JQ1/DMSO)	p-value	FDR
PLXNA2	0.598512521	1.80236E-05	0.003504171
MYL9	0.598547093	0.000577862	0.015170514
HYI	0.598699236	1.48102E-05	0.00323775
SEMA5A	0.59960182	0.000102829	0.00656324
SNORD114-28	0.599781945	0.000998372	0.020707819
HOXB2	0.599960739	6.73014E-06	0.002575353
ALKBH3	0.600081351	0.000325527	0.011181154
LAPTM5	0.600175639	0.002188767	0.032380098
MIR199A1	0.600471079	0.001073777	0.02144674
S100A16	0.60090271	0.00021311	0.009159657
RNASEH2B	0.60114017	0.001400776	0.025005798
WDHD1	0.60201305	7.94046E-05	0.005684161
AURKB	0.602260689	1.93235E-05	0.003557359
CDCA8	0.602333053	0.000120898	0.007154733
ZNF738	0.602915058	0.003679922	0.044875369
VPS52	0.603256447	9.60086E-05	0.006301645
MN1	0.603313596	0.001406294	0.025064495
TNF	0.603784935	7.51835E-05	0.005529648
HIVEP3	0.603964922	5.88944E-05	0.004947862
KRT75	0.604045864	0.00119148	0.022783707
GALNT14	0.604089131	8.35163E-06	0.002681373
BCL2	0.604220345	0.001572629	0.026725721
OR7E14P	0.60457225	0.003995026	0.047170515
SKA3	0.604658862	0.00033506	0.011344438
DOCK10	0.604665847	0.000320287	0.011115243
RARB	0.604970486	0.001379588	0.02479727
GPR126	0.605191376	0.000764254	0.017744536
VPS52	0.605296256	2.0306E-05	0.003617099
VPS52	0.605296256	2.0306E-05	0.003617099
HOXB9	0.605829331	0.000708088	0.016948908
MRPL3	0.606348866	2.4814E-05	0.003790462
SNORA66	0.606751076	0.00409566	0.047760795
IFRD2	0.60711708	0.00086665	0.01914593
SLC44A3	0.607131108	0.000219125	0.009256125
ORC1	0.607486113	6.18156E-05	0.005079279
UFSP2	0.607587179	0.002726759	0.037162862
TRIM8	0.607967735	0.00029599	0.010686933
TPM1	0.608057643	0.001559858	0.026677801

(*Continued*)

GENE	fold change (JQ1/DMSO)	p-value	FDR
BACE2	0.608781603	5.61542E-05	0.004873588
ST3GAL5	0.609327602	0.000733323	0.017283826
RUNX2	0.610371709	1.70976E-06	0.002083338
FANCG	0.610550838	0.000152032	0.00796823
MRPL11	0.610610089	7.15218E-05	0.005424023
PHF5A	0.61125517	0.000714687	0.017051063
FAM171A1	0.611290478	6.52053E-06	0.002575353
LOC100506257	0.611492482	0.00255791	0.035836807
SNORD114-9	0.611864174	0.000837201	0.018726828
ALG8	0.611883966	0.000237628	0.009524872
ARMC4	0.612250237	0.001625131	0.027289273
TMEM54	0.612267213	0.002150313	0.032034824
VPS52	0.612422843	7.63948E-05	0.005567586
VPS52	0.612422843	7.63948E-05	0.005567586
NKX2-5	0.612705908	0.000890155	0.019448835
MUS81	0.612765368	0.000241757	0.009608913
BCL2A1	0.61293387	0.000764825	0.017744536
LRRC4B	0.613313523	0.002977598	0.039081976
MYADM	0.613494933	0.001074067	0.02144674
STIL	0.614449635	0.000109025	0.006734555
STAT6	0.614986509	9.88598E-05	0.00641715
CCDC111	0.615523851	0.002372638	0.034032566
CDCA5	0.616390558	0.000154861	0.008014655
SGCD	0.616542962	0.003907284	0.046554855
TOE1	0.616681156	0.000470866	0.013521329
KIFC1	0.61690347	2.25763E-05	0.003702475
CDKL1	0.617964842	0.000397952	0.012386834
ESX1	0.618366185	0.000257055	0.009915481
UROD	0.618654856	0.000108037	0.00671219
NRM	0.618720611	0.001568692	0.026708702
NRM	0.618720611	0.001568692	0.026708702
NRM	0.618720611	0.001568692	0.026708702
NRM	0.618720611	0.001568692	0.026708702
NRM	0.618720611	0.001568692	0.026708702
NRM	0.618720611	0.001568692	0.026708702
NRM	0.618720611	0.001568692	0.026708702
ADCY3	0.618732048	0.002719669	0.037100455
SOX9	0.618799241	9.32132E-05	0.006170138

GENE	fold change (JQ1/DMSO)	p-value	FDR
CHEK1	0.619053786	3.39285E-05	0.004065425
IL27RA	0.619135319	0.001818574	0.028993899
KCNQ5	0.619246909	0.004325889	0.049368843
ATF1	0.619424349	0.000269768	0.010157409
VPS52	0.619886791	9.71448E-05	0.006351967
FEN1	0.620386845	0.000339104	0.011405361
CGB	0.620505828	0.000203956	0.008994373
KIF23	0.620993469	2.6414E-05	0.003800098
DKK1	0.621424058	0.002171082	0.03220999
HEY1	0.621425494	0.000172204	0.008353596
ANP32E	0.621635155	0.000316262	0.01105412
ATAD5	0.621648082	0.000199572	0.00887268
NSMCE1	0.621665318	0.00237058	0.034030358
AFF3	0.621862129	4.80518E-05	0.004656499
XBP1	0.622234374	0.000395646	0.012363843
PRRX1	0.622720495	0.000321212	0.011115243
CHAF1A	0.622858634	0.00018461	0.008547613
SLC29A1	0.623002561	0.000266979	0.010116334
LOC388796	0.623142203	0.001850518	0.029296357
PION	0.623378369	0.000792065	0.018139719
RNLS	0.623464794	0.001794552	0.028764871
LIN9	0.623499367	2.39147E-05	0.003758137
ATL3	0.623644883	1.13902E-05	0.002969775
C1orf133	0.623652088	0.000930519	0.019940702
WHSC1	0.623665057	6.51784E-06	0.002575353
CBS	0.62377458	0.000140231	0.007661461
TROAP	0.623812053	0.001708729	0.027931989
LPP	0.624385959	0.000451717	0.01322017
NFIX	0.624710637	5.11349E-05	0.004718933
KCNN4	0.624995049	0.000232172	0.009450269
FAM125B	0.62505859	0.000245967	0.009685506
TYMS	0.62512936	3.26313E-05	0.003997539
TPX2	0.625229028	2.94585E-07	0.001434447
SNORD4B	0.625341716	0.000503835	0.014003513
RAB27A	0.625513676	0.004377947	0.049773618
TRHDE	0.626170161	0.000183807	0.00854536
LOC728084	0.626284466	0.004257587	0.048902965
PPIL3	0.62645379	0.001174044	0.022619014

(*Continued*)

GENE	fold change (JQ1/DMSO)	p-value	FDR
SPAG5	0.626565251	0.000179209	0.008500179
PLXND1	0.626928723	0.000351919	0.011654627
ADAMTS6	0.627018537	0.002297962	0.033480932
NLGN1	0.627263418	0.003758708	0.045318337
PXMP2	0.627276462	0.0013901	0.024897217
KIFC1	0.6274736	7.38816E-06	0.002575353
KIFC1	0.6274736	7.38816E-06	0.002575353
LOC100509671	0.627899978	0.001865699	0.029435749
ANAPC15	0.628161168	0.001047251	0.021180859
CENPI	0.628447152	6.56273E-05	0.005212917
MCM6	0.628574942	8.97682E-05	0.006061841
SDC1	0.629100901	0.001175841	0.022619607
SASH1	0.629346596	0.001423243	0.025251496
NKAIN4	0.629398946	0.003997199	0.047175395
EXO1	0.629420759	5.89265E-05	0.004947862
ARHGEF2	0.629494931	0.000125814	0.007245724
CCDC58	0.630107549	0.000254768	0.009855625
LRRC6	0.6304891	4.11259E-05	0.004363083
TRIM14	0.630579424	0.002975771	0.03906756
TMEM209	0.631249976	0.000430648	0.012892269
ADRA1D	0.631499428	0.001676356	0.0276813
GSG2	0.631893501	0.003262417	0.041423595
RFC2	0.631943143	0.002237448	0.032831212
MTHFD1L	0.63209063	0.000103985	0.006566977
CD34	0.632093551	0.001520581	0.026303415
MYBL2	0.63243831	1.33513E-05	0.003200149
TRIM47	0.632593221	0.001278005	0.023685722
SPHK1	0.632790568	3.44663E-05	0.004083199
IGKV1OR2-118	0.632913392	0.002072715	0.031347292
IGKV1OR2-118	0.632913392	0.002072715	0.031347292
NMI	0.633365417	0.001650964	0.027508561
TMEM98	0.633516163	0.000682438	0.016692645
PON2	0.633560077	8.27238E-05	0.005816524
PLK1	0.634009633	4.85752E-05	0.004656499
PSMB9	0.634181047	0.000384938	0.012171722
TMEM218	0.635075491	0.00040341	0.012452294
BUB1B	0.635128318	0.000108965	0.006734555
DPPA4	0.635135655	0.002091966	0.031498158

GENE	fold change (JQ1/DMSO)	p-value	FDR
MMP14	0.635508503	9.82037E-06	0.002909054
CAMKK1	0.63594034	0.00060146	0.015518524
ZHX2	0.635972667	0.000205428	0.009011996
HIST1H2AM	0.63609023	0.00035347	0.011677142
PSMB8	0.636162249	0.000804349	0.018265964
LINC00525	0.636456286	0.002271641	0.033205719
TMEM62	0.636927028	7.50329E-05	0.005529648
MME	0.63704624	0.001663574	0.027554071
HIST1H4A	0.637574868	0.000287717	0.010515694
KIF18A	0.637708935	0.000647113	0.016228371
IFITM4P	0.637856294	0.001164933	0.022500077
IFITM4P	0.637856294	0.001164933	0.022500077
CLPP	0.63803317	3.39418E-05	0.004065425
MCM3	0.638569993	2.79962E-06	0.002229464
GTF2IRD1	0.638754446	0.000232846	0.009450269
LRRK1	0.638868096	0.003894166	0.046460503
TMEM48	0.638884333	0.000168886	0.008281033
ZWINT	0.638955191	9.3535E-06	0.00285781
CCNA2	0.63935244	3.47382E-05	0.004083199
IGFBP3	0.639985001	0.000105986	0.006643128
WNT7B	0.640104786	0.003721371	0.045060622
EXOSC8	0.640166905	0.000396237	0.012366146
C1orf106	0.640384369	0.000783552	0.018019283
SPTBN2	0.640727729	0.001516314	0.026302235
PCDHB8	0.640754377	0.002795236	0.037780473
SNORD14A	0.64104165	0.000594557	0.015395971
RAB7L1	0.64104165	0.001617688	0.027241073
RMI2	0.641744086	0.001554186	0.026648798
PDPN	0.641836022	0.003895574	0.046466961
THY1	0.642183128	0.001084081	0.021623948
CTDSPL	0.642702653	0.000763904	0.017744536
EMG1	0.642825917	0.000158656	0.008093871
RBPMS2	0.643302857	0.004381196	0.049783015
RIN1	0.643316234	0.000951347	0.020179123
SPC24	0.643829238	0.001168835	0.022551071
OCIAD2	0.643982475	0.002847724	0.038142997
KIF21B	0.644567491	0.00164565	0.027478921
RASGRP1	0.644568981	0.002486925	0.035191728

(*Continued*)

GENE	fold change (JQ1/DMSO)	p-value	FDR
IQCB1	0.645399038	0.002505218	0.03537347
AADAT	0.645688394	0.000380198	0.012141201
ZNF827	0.645780896	0.000417304	0.012680208
PHACTR1	0.645809245	0.003991349	0.047147865
CUTA	0.64585998	8.33939E-05	0.005837246
MRPL15	0.646157007	0.000249985	0.009755055
KNTC1	0.646401895	0.000111301	0.006820144
SLC25A43	0.646624466	0.00193884	0.030131822
UAP1L1	0.646939781	0.000899839	0.019572685
LOC100133286	0.647016018	0.002626285	0.03644233
C15orf41	0.647336011	9.31191E-05	0.006170138
ECT2	0.647404815	0.000200265	0.008896113
CUTA	0.647475122	0.000745207	0.017469089
SULF2	0.647837252	0.004006492	0.047222704
NGDN	0.647858208	3.20346E-06	0.002230648
C3orf36	0.647901618	0.004089815	0.04773261
WT1	0.647948026	0.00231254	0.033608531
C17orf53	0.648368842	4.18887E-05	0.004378063
EDNRA	0.648433261	8.87877E-05	0.006036779
HIST1H3E	0.64846772	0.00018411	0.008546689
NABP2	0.648478209	1.88657E-06	0.002083338
NFKB1	0.648707489	0.000454717	0.013264723
CA12	0.649121299	0.000698431	0.016860772
ICAM3	0.649253294	0.00264025	0.036541632
LEF1	0.649305799	0.000769207	0.017838483
KY	0.650131442	0.000281334	0.010398818
TDRD3	0.650331255	9.27383E-05	0.006169168
MTX3	0.650358302	0.000288606	0.010541
PIGB	0.650562694	0.001646582	0.027485924
CHCHD4	0.650615305	0.000134841	0.007507549
TCOF1	0.650676941	0.000181085	0.008500497
BRIX1	0.650837823	0.000597005	0.015426321
AP1S1	0.650970167	0.000772423	0.017866697
TAPBP	0.65111006	0.001969055	0.030355038
---	0.651507338	0.003108944	0.040079887
DAZAP1	0.651797926	0.0011289	0.022114809
GGT3P	0.651888291	0.002338043	0.033780343
TAPBP	0.65196511	0.00161398	0.027196979

GENE	fold change (JQ1/DMSO)	p-value	FDR
TAPBP	0.65196511	0.00161398	0.027196979
KDELR3	0.652302622	0.001143327	0.022244966
MED24	0.652579995	6.25785E-05	0.005106958
KIF14	0.653391685	0.000147236	0.007848115
PARP3	0.653566828	0.000486955	0.013777711
KIAA0101	0.653601561	0.000224641	0.009329649
ZDHHC6	0.653717852	0.00312137	0.040201416
NEK2	0.653837185	0.000358085	0.011735601
EPB41L4B	0.654066849	0.001467494	0.02578435
MIR4640	0.654106142	0.003606644	0.044262398
RFTN1	0.654422081	0.000832451	0.018659501
CAPN2	0.654470468	1.81035E-05	0.003504171
RAPGEF4	0.654993881	9.07725E-05	0.006098098
LOC100507280	0.655054418	0.000119012	0.007102076
CDK2	0.655066526	5.95385E-05	0.004964659
C15orf52	0.655083175	0.000220401	0.009271914
SORCS2	0.655779784	0.002831076	0.03799905
KIF24	0.655911617	0.00073021	0.017239837
SUSD4	0.656375518	0.000754971	0.017608023
GLI2	0.656704691	0.004287921	0.049114604
DIRAS3	0.657009741	0.002755274	0.037418826
MAZ	0.657088683	0.000452208	0.01322017
BCL3	0.657213187	0.000309524	0.010961525
PSMB9	0.657635461	0.004138925	0.048108745
PSMB9	0.657635461	0.004138925	0.048108745
CWC22	0.658362165	0.000201954	0.008941509
MAML2	0.658499082	0.000414246	0.012619675
PPIH	0.658529511	0.000486418	0.013769944
CCNB1	0.658535597	5.68883E-05	0.004873588
IFITM4P	0.658643636	0.003923225	0.046672189
FLI1	0.659245017	0.001906613	0.029811583
VEGFC	0.659269388	0.001065638	0.021375351
RILPL2	0.659290714	0.000703389	0.016891516
DOC2B	0.659379071	0.001305048	0.02395507
B3GALTL	0.659593917	0.002888609	0.038491103
SLC52A3	0.660011622	0.003632598	0.044483903
SH3KBP1	0.66056999	0.001358693	0.024585945
HRCT1	0.660904321	0.001636151	0.027398555

(*Continued*)

GENE	fold change (JQ1/DMSO)	p-value	FDR
CDCA4	0.66097304	0.000607404	0.015597309
TDP1	0.661102863	6.62267E-05	0.005214096
ZBTB8A	0.661484841	0.001143153	0.022244966
LIMS3	0.661617821	0.000378037	0.01210825
SSX2	0.661903743	0.001874707	0.029511618
SSX2	0.661903743	0.001874707	0.029511618
KIF4A	0.661992449	0.000190635	0.008647792
DIAPH3	0.662494324	0.00081151	0.018343479
NUAK1	0.662901612	4.12581E-05	0.004363083
VRK1	0.663234059	0.00103607	0.021065971
HIST2H2AB	0.66372614	4.15201E-05	0.004363083
SH2D4A	0.663729207	0.001415152	0.025157893
PRDM6	0.663977687	0.001895178	0.029726503
TMEM97	0.664367467	0.000162669	0.008189506
PTHLH	0.6645563	1.44719E-05	0.00323775
HPS4	0.664603901	3.97589E-05	0.004332831
SNAPC1	0.66477437	0.000261303	0.009993069
PSMC3IP	0.664818914	0.001972026	0.030374639
MEST	0.665468985	7.2882E-05	0.005470951
DUS4L	0.665547405	0.000889093	0.019448835
TAPBP	0.665862716	0.000381544	0.012162452
SMOX	0.665893486	0.000540272	0.014566311
ARSD	1.499543458	0.001465272	0.025775423
PRAF2	1.49991769	0.001714274	0.02794595
ANAPC16	1.500500015	0.001410633	0.025109679
TNIK	1.500597091	0.000258112	0.009925871
CEP104	1.500860615	0.000390488	0.012272447
NLRP1	1.502803797	0.000349924	0.011596681
TMEM38B	1.503578301	0.000464026	0.013434938
TAF12	1.503651257	0.001054925	0.021255886
VPS13D	1.504172473	0.003023375	0.039422251
AFF2	1.505698937	0.001180573	0.022663366
ANO8	1.505782433	0.000161129	0.008138879
NDST2	1.50588333	0.000567058	0.015007996
HERC2	1.505970316	0.00280305	0.037856708
NIT1	1.506057307	0.002814421	0.037895733
LTA4H	1.506666383	0.000528292	0.014385694
BCDIN3D	1.507307048	0.000973439	0.020431387

GENE	fold change (JQ1/DMSO)	p-value	FDR
SESN1	1.507735471	0.001318122	0.024153707
TMCC2	1.508261588	0.004282681	0.049075552
PIK3R3	1.508327801	4.00146E-05	0.004347483
ZNF2	1.508421898	0.003999589	0.047193214
FAM195B	1.508780916	0.000364737	0.011866568
DLGAP4	1.511879683	6.51945E-05	0.005201687
SDC4	1.512141695	3.26561E-05	0.003997539
ZCCHC2	1.512630905	0.000819445	0.018441233
INPP5F	1.512816147	0.000268838	0.010137534
LGMN	1.513053849	0.000592969	0.015395971
TPRA1	1.514365378	0.000342204	0.011438873
PARD6A	1.515103832	0.000533192	0.014454796
VAT1	1.515173846	0.000349953	0.011596681
FAM53C	1.515194851	0.000318895	0.011101607
DYNLL2	1.515194851	0.000439185	0.013067582
GTF3C1	1.515369903	0.000440353	0.013090585
NXN	1.516588828	5.98573E-05	0.004983492
LMLN	1.51801916	4.13134E-05	0.004363083
PRRC2B	1.51825768	2.08056E-05	0.003622055
ERV3-1	1.518629565	0.000777392	0.017940008
ID1	1.520040749	9.83934E-05	0.006394617
LRRC28	1.520072358	0.000467402	0.013480739
EPHA2	1.520430636	1.11652E-05	0.002963198
G6PD	1.520887387	0.000729161	0.017225867
SKI	1.521590348	0.000368635	0.01195711
NELL2	1.521607926	0.000720572	0.017110234
DMXL2	1.522518755	0.000160954	0.008138879
NRIP3	1.522652436	0.000123785	0.007216119
HTT	1.523166161	0.000154612	0.008010587
CCDC144A	1.523257665	0.001683894	0.027771561
A1BG-AS1	1.524056795	2.25661E-05	0.003702475
ERCC6L2	1.524342048	0.000183365	0.008541687
ASCC1	1.52434557	0.000982476	0.020529001
FAM220CP	1.524803497	0.0029611	0.038960809
LOC100506720	1.525409581	0.002006634	0.030722357
ZNF75A	1.525465973	1.34925E-05	0.003201006
LRBA	1.52697171	6.74914E-05	0.005259719
EIF2C1	1.527222222	0.000444115	0.013126854

(*Continued*)

GENE	fold change (JQ1/DMSO)	p-value	FDR
HUWE1	1.527373961	0.000207793	0.009061273
JAK1	1.527885749	0.00015005	0.007902977
MAN1A1	1.528111696	0.000273683	0.010209303
SEMA4F	1.531533227	0.002519278	0.035471672
CTR9	1.531770331	0.000341526	0.011437601
ZKSCAN1	1.532057028	0.00058336	0.015257567
C3orf23	1.532318996	0.002095858	0.031530196
KDM4C	1.532474782	0.000487211	0.013777711
CYP2U1	1.533165387	7.74184E-05	0.005594262
SLC27A4	1.533207896	0.000155237	0.008015355
PCNX	1.533328345	0.000883847	0.019366254
UBE2D3	1.534210741	0.001189541	0.022778436
ZYG11B	1.535061724	0.003057454	0.039722936
MALAT1	1.535778334	0.000449822	0.01322017
MIB1	1.536115469	0.0004975	0.013936498
PHLPP2	1.536406529	4.86978E-05	0.004656499
EYA4	1.536537879	2.86857E-05	0.003810686
TRAPPC9	1.536605334	3.03793E-05	0.003915497
MSX2	1.53673315	0.001270069	0.023579394
PELI1	1.536860978	6.03737E-05	0.004995458
CLCN5	1.53690714	0.000289272	0.01055095
EPS8	1.536924895	0.000195604	0.00875434
CA8	1.537205454	0.002336031	0.03376037
P4HA1	1.537716983	0.001249709	0.023453967
CASP3	1.538061652	0.000303738	0.010871509
ATF7IP2	1.538264224	0.001496028	0.026059952
C10orf12	1.538264224	0.000127378	0.007312234
KIAA1161	1.538736998	0.000608915	0.015599089
SECISBP2L	1.539092563	2.51358E-05	0.003800098
AGRN	1.539341507	0.000264374	0.010060284
GUSBP4	1.539430426	0.002997728	0.039237256
BTD	1.540497848	0.002089149	0.03147342
ANKRD50	1.540633107	0.00187483	0.029511618
WDR44	1.540771939	0.000161208	0.008138879
ENPP5	1.540789738	0.000420948	0.012750341
ZXDC	1.541601628	0.001593093	0.026962395
PCBP3	1.541744109	0.001652901	0.027508561
ERC1	1.541904415	0.000398472	0.012390749

GENE	fold change (JQ1/DMSO)	p-value	FDR
KLHDC2	1.544311016	0.000422301	0.012777942
BCAS3	1.54460363	0.001658947	0.027520966
KIF21A	1.544896298	0.000573025	0.015087487
AKAP9	1.544978398	0.00169141	0.027834692
CD24	1.545638926	0.000631721	0.016022226
PYGB	1.546871476	6.42907E-05	0.005183571
AMBRA1	1.547379072	0.000412318	0.012604611
MIR3160-1	1.547994129	0.002573694	0.036020295
AARD	1.548358988	0.003369792	0.042293572
BTBD9	1.548978014	0.001713859	0.02794595
PLEKHM1	1.548992329	0.003683853	0.044879787
PITPNM1	1.549160548	1.49155E-05	0.00323775
TOM1L2	1.549246455	4.34567E-05	0.004472203
KCTD21	1.549976849	0.003691216	0.044879787
USP25	1.55000908	0.000152261	0.007972439
EIF5A2	1.550048474	3.96128E-05	0.004332831
IL17RD	1.550610852	0.000608181	0.015599089
SLC26A6	1.551370564	0.004365242	0.049671303
RNF113A	1.551470932	0.000420378	0.012743388
ARMCX3	1.551478101	0.001842541	0.029212804
INPP4A	1.55250007	6.09346E-05	0.005034099
ATG2B	1.553519123	0.000639758	0.016101958
SH3RF1	1.553842201	0.000536566	0.014507846
KLC2	1.554269487	0.000312622	0.010993665
ZBED6	1.554926801	0.000715854	0.017051063
ACOX1	1.554966321	8.62192E-05	0.005948537
TTBK2	1.555976207	0.000321456	0.011115243
VPS13A	1.557019127	0.000724144	0.017149483
RNF123	1.557123457	0.001060477	0.02132768
ATRN	1.557821571	0.000137464	0.007582724
ZBED6	1.564472898	0.000784675	0.018019283
NAP1L3	1.564805486	0.001370196	0.024694386
NAP1L5	1.564921185	0.003487294	0.043251964
KIAA1958	1.564968191	0.001035324	0.021065971
TMEM175	1.567128339	0.000130028	0.007393119
PIK3R1	1.568599088	2.61448E-05	0.003800098
PSKH1	1.568762187	0.000879425	0.019312505
TNFRSF12A	1.56931685	1.44161E-05	0.00323775

(*Continued*)

GENE	fold change (JQ1/DMSO)	p-value	FDR
LCMT2	1.570024059	0.000239325	0.009558746
POTEG	1.570111122	0.00388201	0.04636706
TBC1D24	1.572594449	0.000132611	0.007468702
SGPP1	1.573499442	0.000278194	0.010327673
DENND4A	1.574335842	0.000662563	0.016469467
TEP1	1.574765124	0.000134334	0.00750002
CIRBP	1.575154489	6.9372E-07	0.001586766
RRNAD1	1.57728861	0.001837719	0.029160396
HAGH	1.577325054	3.39689E-05	0.004065425
USP20	1.578787133	0.000403123	0.01245227
TRIM36	1.57901696	0.000855893	0.019023014
GLS	1.579987707	0.000153146	0.00799833
AASDH	1.581452254	0.00014975	0.007901048
VPS39	1.581927337	0.000211548	0.009147233
GLRB	1.582431811	0.000311077	0.01098026
RABGAP1	1.58244278	0.000189716	0.008647792
TOB1	1.583225404	0.00053951	0.014566311
LOC100507556	1.583324174	0.000873285	0.019255822
UBR4	1.584722244	0.000425361	0.012827098
KIAA1109	1.584810122	0.001321105	0.024183574
ARSK	1.585861376	4.28075E-05	0.004448081
CCNYL1	1.585949317	3.80095E-05	0.004277602
GREB1L	1.586165526	0.001164899	0.022500077
KCTD11	1.588347594	0.000104917	0.006618041
BBS2	1.590051323	0.00196415	0.030340487
CYP2R1	1.591683327	0.000706319	0.016921674
CLGN	1.592231379	0.00024871	0.009733638
PAPD5	1.593460584	2.21352E-06	0.002210921
INTS5	1.59390613	0.000125233	0.007226592
FITM2	1.59429286	0.000721691	0.01711919
MARCH2	1.594480735	0.001523676	0.026319245
MFGE8	1.594576523	0.002754913	0.037418826
MERTK	1.595092402	0.000371802	0.011994194
ETHE1	1.595343032	0.003194339	0.040798207
GDE1	1.595376206	1.15176E-05	0.002969775
CTSL2	1.596198419	0.001204753	0.022927325
MYO6	1.596515618	0.000234483	0.009452838
IPO13	1.596611528	0.003497493	0.04333836

GENE	fold change (JQ1/DMSO)	p-value	FDR
FAM149B1	1.596891914	0.001394547	0.024943136
TOR1B	1.597043195	0.000135239	0.007521898
CCNDBP1	1.59719818	0.000445248	0.013145849
RAB9A	1.597460214	0.000693871	0.016832731
PARK2	1.597718599	0.002027646	0.030938047
CKB	1.598039793	6.40366E-07	0.001586766
PKP2	1.599277177	0.000199168	0.008867184
TSPAN31	1.601492084	6.61736E-05	0.005214096
RABIF	1.604128817	0.000165289	0.008255131
GPR125	1.604974082	0.000204175	0.008994373
TAOK2	1.60535608	0.000228838	0.009389169
MPP7	1.605363498	0.000204259	0.008994373
PCYOX1	1.606276215	0.000123016	0.007200599
AGTPBP1	1.606673372	0.000919723	0.01984418
AKAP13	1.609036069	0.000193433	0.008708058
LPPR2	1.610036431	1.7994E-06	0.002083338
CYP7B1	1.61035266	0.001117282	0.021999746
C17orf51	1.610799206	0.000603578	0.015558674
STIM2	1.611715014	0.000480228	0.013659621
KIAA1033	1.612255063	6.44E-06	0.002575353
NCOA3	1.612445054	1.57725E-05	0.003303415
XPR1	1.613145608	2.00309E-05	0.003617099
JMY	1.613872567	0.000673607	0.016599178
ALKBH5	1.614077666	3.6298E-06	0.002289635
SPRY2	1.61469312	0.000176459	0.008455051
PTPRG	1.614715505	2.78852E-05	0.003810686
TMEM50B	1.614939367	0.001042674	0.021138199
SLC23A2	1.616130091	0.000133943	0.007499328
KLHL7	1.617609453	0.000136274	0.007545515
ABCD3	1.618521653	0.000368024	0.011951752
EEFSEC	1.619004131	2.67907E-05	0.003800098
TRIM66	1.619019094	0.00219845	0.032471525
MARCH3	1.620145445	0.000178909	0.008500179
CEND1	1.620239031	0.000332777	0.011292724
PLA2G15	1.620512333	0.000715233	0.017051063
TP53INP2	1.621193916	0.001022673	0.020939905
CYR61	1.621272579	2.06676E-06	0.002172813
PC	1.621478619	0.000773109	0.017874853

(*Continued*)

GENE	fold change (JQ1/DMSO)	p-value	FDR
H6PD	1.621606002	0.000507595	0.0140432
CLTCL1	1.621875787	9.45379E-05	0.006234734
PLXNA3	1.623086626	0.00267716	0.036833792
METTL12	1.623217885	0.000227207	0.009352854
ZZEF1	1.62355921	3.00328E-05	0.003908419
HIST2H2AA4	1.624752536	0.001479407	0.025893494
DENND4C	1.627220771	7.67663E-05	0.005572683
ORAI3	1.627292206	0.000345208	0.011496284
PRKAR2B	1.628349065	1.88647E-05	0.003549013
PIM2	1.629259793	0.000501981	0.013986545
METTL23	1.630174797	0.000257661	0.009925871
OAT	1.630687122	0.000135981	0.007542254
CASP9	1.631320216	0.000686207	0.016746637
CSGALNACT2	1.631527532	0.000518211	0.014224475
TGFB1	1.632300489	0.000150627	0.007925582
PTGER2	1.632311804	0.0012602	0.023526352
RAD54L2	1.632753122	2.67175E-06	0.002229464
RAB3A	1.635067279	0.000882053	0.019342755
CCDC87	1.635520678	0.001033433	0.021041232
LCOR	1.638679068	0.000503165	0.013992841
MOAP1	1.638785084	0.001689142	0.027823879
AMIGO1	1.640311713	0.000804674	0.018265964
WDR7	1.640387513	5.12837E-05	0.004724533
BCORL1	1.640868926	0.000795391	0.018139719
PMAIP1	1.641168459	5.3092E-05	0.004772128
ULK1	1.641240507	0.000354803	0.011692362
ALDOC	1.641653895	0.000376032	0.012072879
TRAF4	1.642814971	0.000194428	0.008731084
STRADA	1.642837746	6.82095E-05	0.005307966
ABCC5	1.644163001	0.001354754	0.024539813
SEMA6C	1.645284035	0.000596079	0.015417254
YPEL5	1.648766008	0.000178588	0.008500179
KIF3A	1.651533996	0.000207559	0.009061273
GNPTAB	1.65159505	0.003671819	0.044814914
PIGZ	1.652828077	0.000358699	0.011742581
MAPT	1.654214898	0.001621385	0.027257236
SAMD4A	1.655273945	0.001652048	0.027508561
KIAA0913	1.657593212	0.000840742	0.018776995

GENE	fold change (JQ1/DMSO)	p-value	FDR
ATP10D	1.659892717	0.000341792	0.011438873
UBR3	1.660383691	0.000864945	0.019128163
MLL3	1.661565693	2.7937E-05	0.003810686
KIAA1919	1.664036025	0.000371087	0.011994194
DNAJC6	1.664351323	3.59339E-05	0.004153722
C4orf32	1.664924397	5.69011E-05	0.004873588
SCAND3	1.66505904	0.001377103	0.024777225
NOP14-AS1	1.66545149	0.000579219	0.015186301
TPI1P2	1.667966138	0.001803341	0.028845386
TSPAN7	1.669512236	0.001780686	0.028679796
GJA3	1.673493948	0.000442523	0.013101466
GADD45B	1.674066301	0.0001707	0.008305283
FBXO33	1.674542122	0.000141239	0.007664789
MAPRE3	1.674867151	4.48842E-05	0.004589186
SLC16A10	1.674867151	0.000306381	0.010907855
GDPD1	1.675331587	0.004001372	0.047197196
C6orf226	1.676225989	0.004057566	0.047573697
KAT2B	1.677500659	0.000122923	0.007200599
CTC1	1.678248865	0.000392347	0.012302029
RN7SKP4	1.678543587	8.40434E-05	0.005852149
TTC12	1.678679331	0.001783895	0.028688262
SLC22A17	1.679773445	0.000101613	0.006524772
RFX3	1.680153836	0.000455868	0.013283845
MLLT4	1.680755651	0.000247759	0.009717699
LOC100506730	1.681276103	0.000319332	0.011103517
TECPR2	1.681952154	0.000439117	0.013067582
MFSD9	1.68269457	0.000621244	0.015833019
ASNS	1.683289516	9.80012E-07	0.001684565
WDR26	1.685126232	4.13855E-05	0.004363083
SLC17A5	1.687046805	0.000747835	0.01750182
SLC7A5	1.687167644	2.80812E-05	0.003810686
C5orf42	1.687861664	0.000470483	0.013518696
KDM4B	1.687959162	0.000186093	0.008594099
LRP6	1.689273978	0.000358736	0.011742581
GXYLT2	1.690882801	7.94567E-06	0.002661222
LOC100134229	1.69157444	0.00261724	0.036346494
MMP15	1.697302056	0.003549515	0.043831033
MAP1A	1.698114022	0.003177853	0.040626358

(*Continued*)

GENE	fold change (JQ1/DMSO)	p-value	FDR
KIF5C	1.698785069	0.002180183	0.032282483
NFAT5	1.699511354	2.01578E-05	0.003617099
IRS2	1.700194738	0.000417052	0.012680208
HOMER1	1.700253663	0.000116909	0.007021906
ACVR1C	1.702101023	0.001648911	0.02749325
ALDH3A2	1.702750041	0.00046008	0.013362174
H1FX	1.705407689	0.00030032	0.010792398
OGDH	1.705659889	1.17065E-05	0.002969775
BAIAP2	1.70656654	4.87495E-05	0.004656499
CNNM3	1.707359268	6.40868E-06	0.002575353
FRRS1L	1.71151033	0.000604592	0.015569996
ROBO1	1.71186231	0.000417484	0.012680208
KIAA0319L	1.712428003	0.000187662	0.008647792
DGCR2	1.71344118	8.82294E-05	0.006033923
WNK3	1.713504523	0.001498584	0.026095998
SLC12A6	1.713999474	3.14642E-05	0.003996833
SYNM	1.714161849	0.000156886	0.008049528
ZNF554	1.717686401	0.001660577	0.02753858
USP53	1.718412826	0.000905545	0.019617215
FBXW5	1.719052176	9.43418E-06	0.00285781
PTPN13	1.725705959	0.000737079	0.017340924
RDH10	1.725885394	0.00020991	0.00909107
APLP2	1.727317551	1.83398E-05	0.003510063
AHR	1.733594723	0.000139928	0.007661461
LOC100132273	1.737002704	0.001518978	0.026303415
FAM173A	1.742964679	0.002483053	0.035155493
SIPA1L2	1.745322127	0.002087997	0.031464906
PLD3	1.745447141	2.43225E-05	0.003758137
PPM1L	1.745467305	0.000833952	0.018677529
SOCS1	1.746286173	0.00172683	0.028090851
FAM102A	1.747501063	0.002576548	0.03604706
MT1P3	1.748219901	0.000103835	0.006565237
TIAM1	1.750802885	4.16316E-05	0.004363083
AHCYL2	1.755972194	1.1657E-05	0.002969775
PGPEP1	1.75744556	0.001274039	0.02363673
SLC2A13	1.758119743	3.51324E-05	0.004086104
MYO9A	1.758140053	0.002100875	0.031561394
ADI1	1.763371767	4.87508E-06	0.002398047

GENE	fold change (JQ1/DMSO)	p-value	FDR
SLC25A4	1.763705888	0.000208187	0.009061273
ZC3H10	1.76469232	0.000400458	0.012423887
MTHFR	1.764810566	0.003155876	0.040480527
GLCCI1	1.767066908	0.000467248	0.013480739
ERCC6	1.767189396	8.28106E-06	0.00268001
SRXN1	1.767262893	4.54251E-06	0.002376493
FBXL20	1.76747115	0.000126818	0.007287889
CNKSR3	1.767679433	0.001347068	0.024491353
POTEM	1.773886045	0.000277603	0.010321942
CPT2	1.777960574	0.002806695	0.037872472
N6AMT1	1.781830371	0.000201932	0.008941509
SYNJ1	1.782015641	0.000160352	0.008133958
SLC30A4	1.782200931	0.000173674	0.00838908
MGC12916	1.78307411	0.001475986	0.025870527
KDM5B	1.78725233	5.68609E-05	0.004873588
VGF	1.78962833	0.000594969	0.015395971
ALAD	1.791022341	0.000658078	0.016418878
EFTUD1	1.791154766	0.001843724	0.029212804
SLC12A2	1.794223982	2.95628E-06	0.002230648
KIF1B	1.794692488	0.000147599	0.007848115
HSPA4L	1.795526153	0.000123573	0.007216119
ZCCHC24	1.796144392	2.76887E-05	0.003810686
PIK3C2B	1.796156842	0.002593077	0.036131239
SEMA6A	1.797688847	0.003755775	0.045302518
NXF1	1.798827278	0.000188261	0.008647792
RGL1	1.80078174	2.33332E-05	0.003745677
FAM214A	1.802813302	3.61533E-05	0.004153722
BRD2	1.802913274	2.39533E-05	0.003758137
PAIP2B	1.811146607	0.00082927	0.018619334
SLAIN1	1.812029781	0.000142735	0.00771474
C1orf38	1.817069162	0.001343665	0.024446314
ACER2	1.818043434	0.000478906	0.013636486
TEC	1.82026269	0.003036713	0.039548079
RHPN2	1.823870545	0.000132412	0.007465336
EGR1	1.824232988	0.000242118	0.009608913
VPS8	1.827249113	0.000747257	0.01749593
CEP19	1.829073859	0.000356305	0.011698947
TXNRD1	1.839308316	3.71755E-05	0.004222964

(*Continued*)

GENE	fold change (JQ1/DMSO)	p-value	FDR
FAM8A1	1.847716393	0.00012992	0.007393119
FAM171B	1.848019526	1.23412E-05	0.003021453
ABTB2	1.848412392	9.15073E-06	0.002836039
C7orf43	1.848438017	4.06231E-05	0.004347483
BCL2L11	1.84850208	0.000384526	0.012171722
CDON	1.850095829	0.000116951	0.007021906
MAP6D1	1.850702926	0.004065516	0.047601699
PLEKHH1	1.851057871	6.4198E-05	0.005183571
NEFL	1.851442828	0.000230753	0.009428262
TCP11L2	1.851973344	0.000134751	0.007507549
C11orf67	1.853480153	0.002727416	0.037162862
ITFG3	1.857608742	0.001161713	0.022470262
TERF2IP	1.857685999	1.17116E-05	0.002969775
CPM	1.869164229	0.000448185	0.013198821
SLC41A2	1.873782282	4.40478E-06	0.002367448
C12orf51	1.874825947	3.28057E-06	0.002255055
MLYCD	1.878272867	0.000103585	0.00656324
PLCG2	1.881191432	2.40303E-05	0.003758137
HEATR5A	1.883278898	3.61266E-06	0.002289635
EXOC6B	1.884079707	5.07218E-05	0.00471326
EVI5	1.886667252	4.10199E-07	0.001586766
MYCBP2	1.887626504	0.000552766	0.014789249
CALN1	1.887635227	0.000168591	0.008281033
BRD2	1.894867101	2.06456E-05	0.003622055
RN5S139	1.897237119	0.00380802	0.04577906
ZSWIM5	1.902434346	0.000247645	0.009717699
BRD2	1.903063015	3.2426E-05	0.003996833
EPB41L5	1.905606184	2.86137E-05	0.003810686
GSTT2	1.910654162	0.003569819	0.04399026
ABCC10	1.911842044	0.000154431	0.008010587
CACHD1	1.918656792	4.90177E-05	0.004656499
SLC25A30	1.920692636	0.000286091	0.010499642
C14orf118	1.930047919	0.000174702	0.008408435
BRD2	1.930877537	1.11455E-05	0.002963198
DPP3	1.936068503	0.000140039	0.007661461
FAM89B	1.937410949	0.000106515	0.006656194
ZNF468	1.93850797	0.000169879	0.008283632
BEND6	1.939991054	0.003875691	0.046301899

GENE	fold change (JQ1/DMSO)	p-value	FDR
ZFYVE1	1.945206521	4.57311E-05	0.004592806
ACADSB	1.945296411	0.000130785	0.007419777
HIST2H4B	1.946069634	0.0015725	0.026725721
GSTT2B	1.948031041	0.000325031	0.011178439
LOC100129033	1.952902604	0.000721907	0.01711919
REEP6	1.955548522	0.00066763	0.016518836
SERINC5	1.964238345	3.03662E-05	0.003915497
AMIGO3	1.973336057	0.001517066	0.026303415
EPHX1	1.981404362	1.67267E-05	0.003403309
SERPINB9	1.982306437	0.001098923	0.021766007
ZKSCAN2	1.984611566	0.000333871	0.011322683
CAPN5	1.984905055	5.60774E-05	0.004873588
FAM59A	1.991276185	0.000267596	0.010125402
DENND2C	1.99943632	0.000441221	0.013090585
MGAT4A	2.001137084	0.000134268	0.00750002
KDM1B	2.004084492	3.7162E-06	0.002307559
HERC1	2.009360841	0.000310243	0.010972493
ERO1LB	2.010113085	0.000523052	0.014293822
MRPS17	2.010410345	0.000715129	0.017051063
RETSAT	2.012645848	1.30501E-05	0.003151834
SLFN5	2.014814008	4.15599E-06	0.002359839
SORBS1	2.015614863	0.000572994	0.015087487
RALGAPA1	2.017003145	2.36811E-05	0.003758137
ZDBF2	2.017320068	0.000114976	0.006981504
TMEM106A-AS1	2.018583594	0.001810044	0.028909481
GMPR	2.02809233	0.000234015	0.009450269
SESN2	2.02824697	0.000799162	0.018182987
ZER1	2.033361415	1.97981E-05	0.003598355
JHDM1D	2.038931537	0.001387197	0.024897217
GATM	2.040156747	4.59492E-05	0.004592806
TOB2P1	2.040637608	0.000383629	0.012171722
SPATA7	2.047514024	0.000705899	0.016919172
ARRDC3	2.047698532	1.85787E-07	0.001272452
PAG1	2.053706475	0.000456842	0.013304996
B3GALT1	2.054650961	0.000363305	0.011841534
JUP	2.060960231	1.44221E-06	0.002083338
LOC653562	2.061684157	0.00202439	0.030905956
MXD1	2.06665848	7.21654E-06	0.002575353

(*Continued*)

GENE	fold change (JQ1/DMSO)	p-value	FDR
FOS	2.069147751	0.000946949	0.020147843
KLHL24	2.070893459	3.26285E-05	0.003997539
ADARB1	2.081244799	0.000101341	0.006515108
DYRK1B	2.085683143	0.000586105	0.015299509
TLCD1	2.086333803	0.000159184	0.008103391
NEU1	2.086550734	2.69324E-05	0.003810117
SLC9A1	2.086926803	5.44373E-05	0.004815292
CDKN1A	2.092953124	0.000501651	0.013986545
WDR47	2.094583406	3.39176E-06	0.002255733
PNPLA8	2.099724431	1.1219E-05	0.002963198
DNAJC28	2.100253299	0.000304288	0.010876673
LOC100506670	2.105777977	0.002312988	0.033608531
ODZ1	2.105831496	0.002119047	0.031735919
ZFP36	2.108665124	0.000745459	0.017469089
SYN1	2.114568554	0.001296315	0.023881594
GLCE	2.120562003	2.14038E-05	0.003631669
ENC1	2.126611748	1.73219E-06	0.002083338
SPTAN1	2.130738186	1.47875E-05	0.00323775
NEU1	2.134433675	1.93551E-05	0.003557359
POLR2A	2.138219557	2.35107E-05	0.003751706
NEU1	2.140216385	5.91525E-05	0.004947862
JUND	2.142784353	5.6357E-05	0.004873588
DOPEY2	2.146307324	1.59933E-05	0.003326561
TNFSF9	2.14749782	0.000159896	0.008126203
SLC9A3R1	2.149697011	2.53187E-05	0.003800098
TARSL2	2.151754272	0.000702636	0.016889235
ROPN1L	2.156841259	0.000917909	0.019828979
KCTD13	2.16298451	2.59257E-06	0.002229464
MAP3K9	2.164009251	0.001330455	0.024296664
NUDT7	2.166250376	9.29344E-05	0.006170138
CDO1	2.166986251	0.000464748	0.013440342
UNC13B	2.168012889	0.000107167	0.006677776
PNMA1	2.169035001	5.81677E-05	0.004942595
ATF3	2.169435961	0.00393569	0.046747896
GDF11	2.177189022	6.63225E-05	0.005214096
EDEM2	2.179655301	0.000265168	0.01006906
NBEA	2.182648783	0.000111189	0.006820144
ABCB1	2.198927441	0.000792137	0.018139719

GENE	fold change (JQ1/DMSO)	p-value	FDR
RSPH3	2.202690302	5.20917E-05	0.004750001
KIAA0513	2.211205843	0.000228876	0.009389169
PNPLA2	2.21187522	3.12978E-05	0.003985972
HOXA1	2.236108202	1.34015E-05	0.003200149
TM7SF2	2.244929284	0.000251282	0.009765637
BRD2	2.248537067	1.36065E-06	0.002083338
ATP8A1	2.251557518	0.002492951	0.035248831
BAMBI	2.257553222	0.000268149	0.010132026
GATS	2.260157535	0.001110299	0.021915894
LIFR	2.271224001	0.000115739	0.006996142
RALGAPA2	2.274737388	2.61435E-05	0.003800098
NPTXR	2.277251029	5.3306E-05	0.004772128
GATSL1	2.279540961	5.69438E-06	0.002522548
TBC1D8	2.284534136	6.72935E-05	0.005259719
ZSWIM6	2.285236271	8.72301E-06	0.002767465
HIST2H2BC	2.288300733	0.000245252	0.009685506
GCH1	2.310230284	3.70578E-05	0.004218531
H1F0	2.314408189	0.000105071	0.006619967
ELOVL4	2.346873369	7.6274E-07	0.001586766
SAMHD1	2.34707943	8.31558E-07	0.001647978
IRF7	2.358113784	0.000130704	0.007419777
C4orf34	2.367120802	0.000654547	0.016376503
HIST2H2BF	2.370207447	0.000944873	0.020127634
FNBP1	2.384863454	1.78826E-06	0.002083338
CITED2	2.398423903	2.48424E-06	0.002229464
ERN1	2.402722441	0.000581749	0.015237731
CXADR	2.437282252	0.002911311	0.038650357
INA	2.450257435	3.17039E-05	0.003996833
CNNM4	2.455669943	7.56624E-05	0.005549646
PNRC1	2.4689883	5.79759E-05	0.00493696
C14orf37	2.472207777	3.24262E-05	0.003996833
TESK2	2.473927691	0.000129588	0.007383762
RORB	2.474682316	2.19823E-05	0.003661641
DGAT1	2.49901576	2.89555E-06	0.002229464
DLX2	2.509946593	8.87669E-06	0.002799656
SHISA2	2.530063042	5.31054E-05	0.004772128
CTH	2.567652563	7.1101E-06	0.002575353

(*Continued*)

GENE	fold change (JQ1/DMSO)	p-value	FDR
MKNK2	2.576388219	9.76118E-06	0.002909054
FLNC	2.586724965	3.03387E-05	0.003915497
ITPR1	2.598045445	9.88347E-06	0.002911659
CLU	2.644551981	0.000671873	0.01658555
ANGPTL4	2.647339707	1.45441E-05	0.00323775
MIDN	2.656806824	7.30513E-07	0.001586766
ATP1B2	2.713917127	0.000793473	0.018139719
IL11	2.785436319	1.55394E-05	0.003303415
GPR50	2.790035219	7.34245E-06	0.002575353
TP53INP1	2.797458379	0.000429854	0.012882885
CHMP1B	2.825618447	4.26669E-05	0.00444208
TMEM2	2.865261208	3.54202E-06	0.002288102
SERTAD1	2.870761212	4.21391E-06	0.002359839
HIST2H4B	2.885630679	1.02479E-05	0.002925673
HIST2H4B	2.885630679	1.02479E-05	0.002925673
TMEM47	3.244058869	0.000252685	0.009803337
DCXR	3.296556939	1.4402E-05	0.00323775
HIST2H4B	3.315768612	0.000558348	0.014886596
SLIT2	3.381078081	0.001782085	0.028685095
EFR3B	3.392863368	8.69909E-06	0.002767465
LINC00052	3.417615274	0.001043959	0.021138199
TEX14	3.459803255	0.000116725	0.007021906
HIST1H2BD	3.54615763	0.000183922	0.00854536
PEG10	3.571747266	3.06944E-06	0.002230648
HIST1H2AH	3.636145676	3.46836E-05	0.004083199
DHRS2	3.664111316	9.5058E-05	0.006261333
LOC729974	3.70070465	7.69119E-05	0.005572683
SERPINI1	3.854668159	0.000226396	0.009352854
HEXIM1	3.912290051	5.7398E-06	0.002522548
HIST1H2BC	4.593604556	0.000304241	0.010876673
HIST2H2BE	4.597724433	3.19589E-05	0.003996833
HIST1H2BC	5.014896757	6.56385E-06	0.002575353
FAM46C	5.410880327	8.09033E-06	0.002661222
SESN3	5.861134726	0.000874637	0.019274728

Supplementary Table S2. Summary statistics of cell cycle analysis in multiple medulloblastoma cell lines.

Cells/Treatments	G1	S	G2/M
Daoy DMSO	50.67 ± 3.19	15.39 ± 1.73	33.94 ± 1.46
Daoy JQ1 300nM	79.416 ± 0.273 (*)	3.736 ± 0.421	16.813 ± 0.238
D283 DMSO	23.47 ± 0.29	19.88 ± 1.26	55.70 ± 1.50
D283 JQ1 300nM	30.373 ± 0.493 (*)	14.093 ± 0.655	53.216 ± 0.384
D425 DMSO	33.715 ± 0.495	28.96 ± 0.89	37 ± 0.47
D425 JQ1 300nM	39.393 ± 0.796 (*)	28.636 ± 1.582	31 ± 0.83
D458 DMSO	35.85 ± 1.37	43.54 ± 3.48	18.72 ± 2.62
D458 JQ1 300nM	46.86 ± 1.96 (**)	26.58 ± 1.57	23.266 ± 2.52

*$P < 0.01$ and **$P < 0.05$ increase in G1 for cell lines treated with 300 nM JQ1 *vs*. their respective DMSO treated control
